# Epigenetics: the master switch of nasopharyngeal carcinoma invasion and metastasis

**DOI:** 10.3389/fimmu.2026.1820374

**Published:** 2026-05-04

**Authors:** Hongpeng Chen, Yimao Wu, Kaiyu Zhang, Zichang Chen, Hongwei Xu, Chunrong Xie, Chunping Lin, Xiaocheng Pan

**Affiliations:** 1 Department of Medical Oncology, Jieyang People’s Hospital, Jieyang, China; 2 Second Clinical Medical College, Guangdong Medical University, Dongguan, China; 3 Second Clinical Medical College, Anhui Medical University, Hefei, China

**Keywords:** biomarkers, DNA methylation, epigenetic regulation, histone modification, metastasis, nasopharyngeal carcinoma, therapeutic targets

## Abstract

Nasopharyngeal carcinoma (NPC) is a metastasis-prone malignancy that is highly prevalent in endemic regions. Epigenetic dysregulation, including aberrant DNA/RNA/protein methylation, histone modifications, and non-coding RNA networks, contributes to NPC invasion and metastasis by reshaping epithelial-mesenchymal transition, tumor microenvironment interactions, immune evasion, and therapy adaptation. This review provides a structured overview of the core mechanisms of epigenetic regulation in NPC, highlighting aberrant methylation (DNA, RNA, protein), histone post-translational modifications (acetylation, phosphorylation, ubiquitination, palmitoylation), and the dysregulation of non-coding RNAs. We also summarize current translational progress, including biomarker development and early NPC-specific epigenetic trials, while highlighting the major barriers to clinical implementation, such as tumor heterogeneity, off-target effects, and insufficient validation. Overall, this review integrates mechanistic and translational evidence to clarify how epigenetic insights may support biomarker-guided and precision-oriented management of NPC.

## 1 Introduction

NPC is a distinctive epithelial malignancy originating from the nasopharyngeal mucosa and is characterized by marked geographic and ethnic disparities (1). Epidemiologically, NPC shows a particularly high incidence in southern China, Southeast Asia, and North Africa, with age-standardized incidence rates exceeding 20 per 100, 000 population in endemic regions (2). This striking distribution is closely associated with genetic susceptibility (3), Epstein–Barr virus (EBV) infection (4), and environmental exposures, including dietary nitrosamines (5). Clinically, NPC is frequently diagnosed at an advanced stage because of its deep anatomical location and the nonspecific nature of its early symptoms, which together contribute to an unfavorable prognosis, especially in patients with distant metastases (6). Metastatic spread remains the leading cause of treatment failure, with 5-year survival declining from more than 80% in patients with localized disease to less than 50% in those with metastatic disease (7).

**Figure 1:**
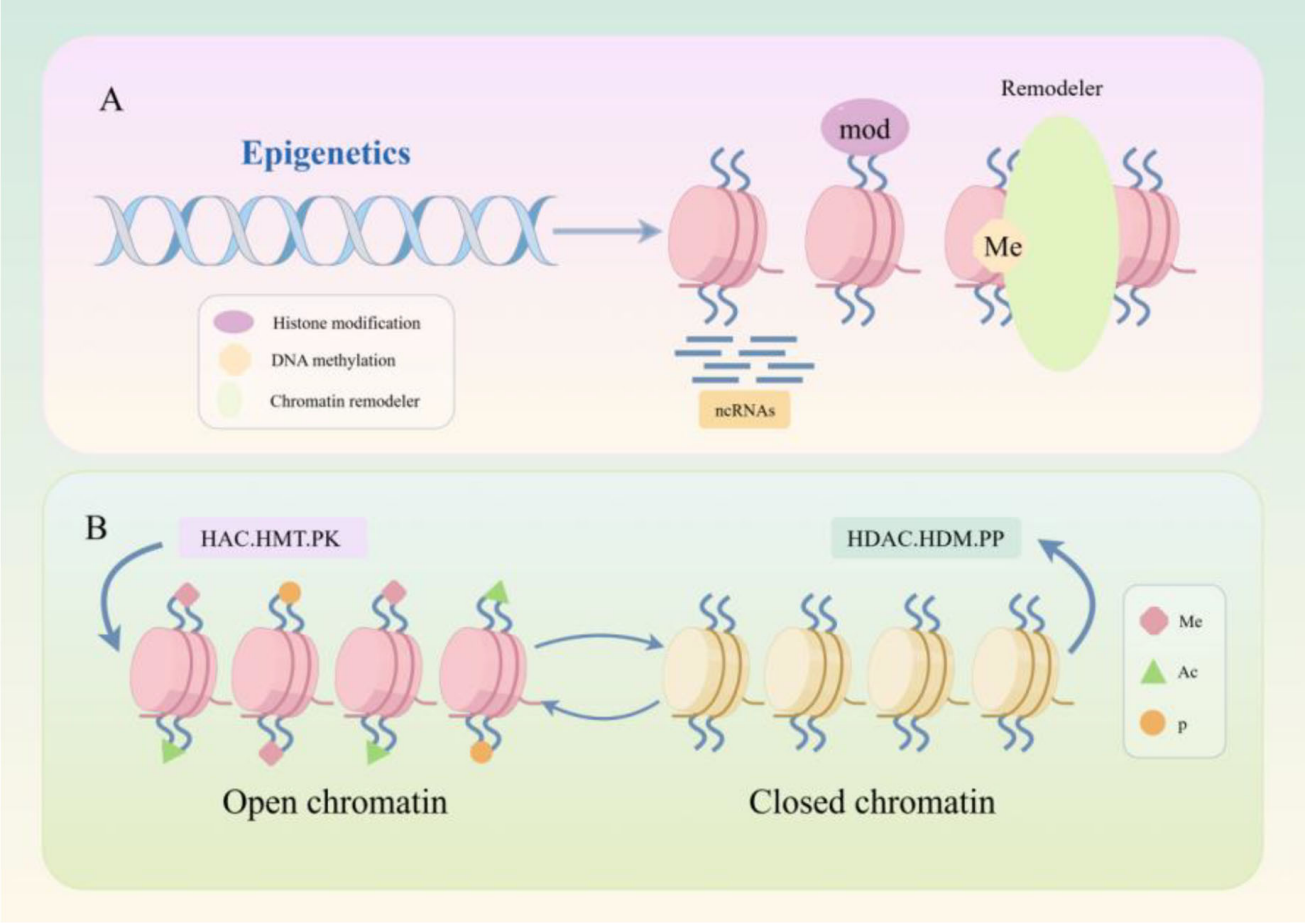
Core epigenetic mechanisms regulating chromatin accessibility in NPC. **(A)** Overview of the three major epigenetic layers discussed in this review: DNA methylation (Me), histone modifications (mod), non-coding RNA (ncRNA) regulation, and chromatin remodelers, which together influence chromatin structure and transcriptional output. **(B)** Dynamic regulation of chromatin states by histone-modifying enzymes. Enzymes such as histone acetyltransferases (HAC), histone methyltransferases (HMT), and kinases (PK) promote an open (transcriptionally permissive) chromatin state, while histone deacetylases (HDAC), histone demethylases (HDM), and phosphatases (PP) drive the formation of a closed (transcriptionally repressive) chromatin state. Key histone modifications include methylation (Me), acetylation (Ac), and phosphorylation (p). The key message of this image is that distinct epigenetic alterations converge on a shared set of malignant phenotypes, including invasion, metastasis, and therapy resistance.

Research into NPC pathogenesis has traditionally focused on genetic mutations, but accumulating evidence indicates that epigenetic regulation functions as a critical upstream driver of invasive and metastatic behavior. Unlike isolated downstream effectors, epigenetic mechanisms operate at the level of chromatin accessibility and transcriptional programming, thereby governing whether broad gene networks related to epithelial-mesenchymal transition (EMT), cell adhesion, migration, immune evasion, and therapy adaptation are activated or repressed (8). In this sense, epigenetic regulation acts as a “master switch” not because it represents a single dominant pathway, but because it integrates diverse upstream inputs—including EBV-associated signals, environmental stress, and tumor microenvironmental cues—and translates them into coordinated phenotypic outputs (9). The core epigenetic mechanisms regulating chromatin accessibility are shown in **Figure 1**. Epigenetic dysregulation reshapes transcriptional programs that govern epithelial–mesenchymal transition, cell motility, tumor–microenvironment interactions, and immune evasion, thereby facilitating invasion and metastasis (10, 11).

Despite substantial progress, several major challenges remain. First, the intricate architecture of epigenetic regulatory networks, together with extensive crosstalk among DNA methylation, histone modifications, and non-coding RNAs, complicates the identification of key regulatory nodes (12). Second, both intertumoral and intratumoral heterogeneity hinder the development of broadly effective therapeutic strategies (13). Third, currently available epigenetic biomarkers, such as methylated DNA loci, still lack standardized detection methods, thereby limiting their clinical applicability (14). In addition, off-target effects associated with broad-spectrum epigenetic agents, including DNMT inhibitors, as well as the emergence of drug resistance, continue to compromise therapeutic efficacy (15).

Recent advances have redirected attention toward several emerging frontiers, including single-cell epigenomics, which enables characterization of heterocellular epigenetic landscapes; epigenetic editing technologies, such as CRISPR–dCas9-based platforms, which offer greater targeting precision; and the role of the tumor microenvironment in shaping epigenetic states (16). Moreover, the interplay between EBV and the host epigenome, particularly EBV-associated DNA hypermethylation, has increasingly been recognized as a critical yet insufficiently understood area of investigation. In NPC, EBV should not be viewed only as an etiologic background factor, but also as an active epigenetic modulator (17). Through persistent latent infection, EBV can reshape host DNA methylation, cooperate with histone-regulatory machinery, and interact with non-coding RNA networks, thereby creating a permissive epigenetic context for invasion and metastasis (18).

Importantly, the available evidence is not fully concordant across studies. The main sources of inconsistency include context-dependent effects of specific regulators, differences between primary, recurrent, and metastatic lesions, variability in assay platforms and normalization pipelines, and cohort-dependent differences in geography, EBV background, treatment exposure, and specimen type (19). Therefore, in addition to summarizing established mechanisms, this review compares convergent and divergent findings, distinguishes association from stronger causal inference where possible, and identifies key evidence gaps relevant to clinical translation. Recent reviews have provided valuable summaries of general epigenetic alterations in NPC or have discussed individual regulatory layers separately. In contrast, the present review is specifically organized around invasion and metastasis, which are the most clinically consequential phenotypes in NPC (20). Most existing reviews summarize epigenetic events in NPC but pay less attention to how different regulatory layers converge on shared metastatic phenotypes. Here, we emphasize epigenetic dysregulation as an upstream organizing layer that coordinates multiple downstream programs related to invasion and metastasis (21). Conceptually, we propose an invasion–metastasis-centered framework in which epigenetic dysregulation is viewed as an upstream organizing layer that connects molecular alterations across DNA, RNA, histone, and non-coding RNA regulation to a limited set of downstream metastatic programs, including EMT, microenvironment remodeling, immune evasion, therapy adaptation, and biomarker-linked clinical stratification (22). In addition, this review proposes a translational perspective by connecting mechanistic epigenetic insights with prognostic stratification and precision epigenetic intervention, thereby providing a metastasis-oriented synthesis that is less explicitly addressed in existing NPC reviews. Taken together, the main novelty of this review lies in reframing NPC epigenetics not as a collection of parallel molecular abnormalities, but as a metastasis-oriented regulatory architecture with direct implications for biomarker development and mechanism-guided therapeutic intervention.

### 1.1 Literature search and study selection

To improve methodological transparency, the literature included in this review was identified through structured searches of PubMed, Web of Science, and Embase. Search strategies combined disease-related terms (“nasopharyngeal carcinoma” or “NPC”) with epigenetics-related terms (“epigenetic regulation,” “DNA methylation,” “RNA methylation,” “histone modifications,” and “non-coding RNA”) and clinically oriented terms (“invasion,” “metastasis,” “biomarker,” “prognosis,” and “therapy”). The search was designed to capture both mechanistic studies and translationally relevant reports related to NPC invasion, metastatic progression, prognostic stratification, and epigenetic therapeutic strategies. We primarily included English-language original research articles and selected review articles that were directly relevant to the scope of this review. Priority was given to studies providing clear mechanistic insight, *in vivo* validation, clinical specimen support, or translational relevance. Recent studies were preferentially used to reflect current progress in rapidly evolving areas, whereas landmark earlier studies were retained when they established foundational concepts, key regulatory mechanisms, or widely cited translational observations in the field. Conference abstracts without sufficient methodological or outcome detail, duplicate publications, studies with only marginal relevance to NPC, and reports lacking adequate experimental or clinical context were not prioritized. Additional relevant articles were identified through manual screening of reference lists from key publications. This review was conducted as a structured narrative synthesis rather than a formal systematic review; however, the above strategy was used to improve coverage, selection transparency, and interpretive rigor. When multiple studies addressed the same regulatory axis, emphasis was placed not only on more recent reports, but also on studies with stronger mechanistic design, greater consistency across models, clearer causal evidence, or higher translational significance.

## 2 Core mechanisms of epigenetic regulation

### 2.1 Methylation modification: multi-level regulation from DNA to proteins

#### 2.1.1 DNA methylation: establishing, maintaining, and demethylating dynamic balance

DNA methylation, predominantly in the form of 5-methylcytosine at CpG dinucleotides, is catalyzed by DNMT family enzymes (23). DNMT1 primarily maintains methylation patterns during DNA replication, whereas DNMT3A and DNMT3B mediate *de novo* methylation (24). DNMT3L lacks catalytic activity but facilitates DNMT3A/3B function (25). Together, these enzymes establish and preserve methylation states that are highly relevant to gene regulation and tumor biology (26, 27)Most functionally relevant DNA methylation events occur at CpG-rich regulatory regions and are interpreted by methyl-CpG-binding proteins that couple methylation marks to downstream transcriptional control (28).

These methylation marks are interpreted by methylation-sensitive binding proteins that link DNA methylation to downstream transcriptional repression or maintenance of epigenetic stability (29–32).

DNA methylation is reversible, and TET family enzymes mediate active demethylation through stepwise oxidation of 5mC, ultimately enabling restoration of unmethylated cytosine (33).

In NPC, aberrant promoter hypermethylation and locus-specific hypomethylation are recurrent events that contribute to invasion, metastasis, and therapeutic vulnerability. In the context of EBV-associated tumorigenesis, persistent viral latency can bias the host methylome toward hypermethylation of tumor-suppressor genes, thereby stabilizing malignant transcriptional programs and reinforcing invasive behavior. This EBV-driven epigenetic reprogramming provides a mechanistic link between a defining etiologic factor of NPC and downstream epigenetic dysregulation.

#### 2.1.2 RNA methylation: the “write-read-erase” mechanisms of m^6^A and m^5^C

RNA methylation, particularly m6A and m5C, is regulated by writer, reader, and eraser proteins and influences RNA fate at the levels of stability, splicing, translation, and degradation. In NPC, the significance of this regulatory layer lies less in the full catalog of these factors and more in how RNA methylation reshapes post-transcriptional programs associated with stress adaptation, EMT, and metastatic progression (34).

Among RNA methylation marks, m6A is the most extensively studied, mainly written by the METTL3/METTL14/WTAP complex (35, 36), whereas m5C is mediated by NSUN and DNMT2 family proteins (37, 38).

**Figure 2:**
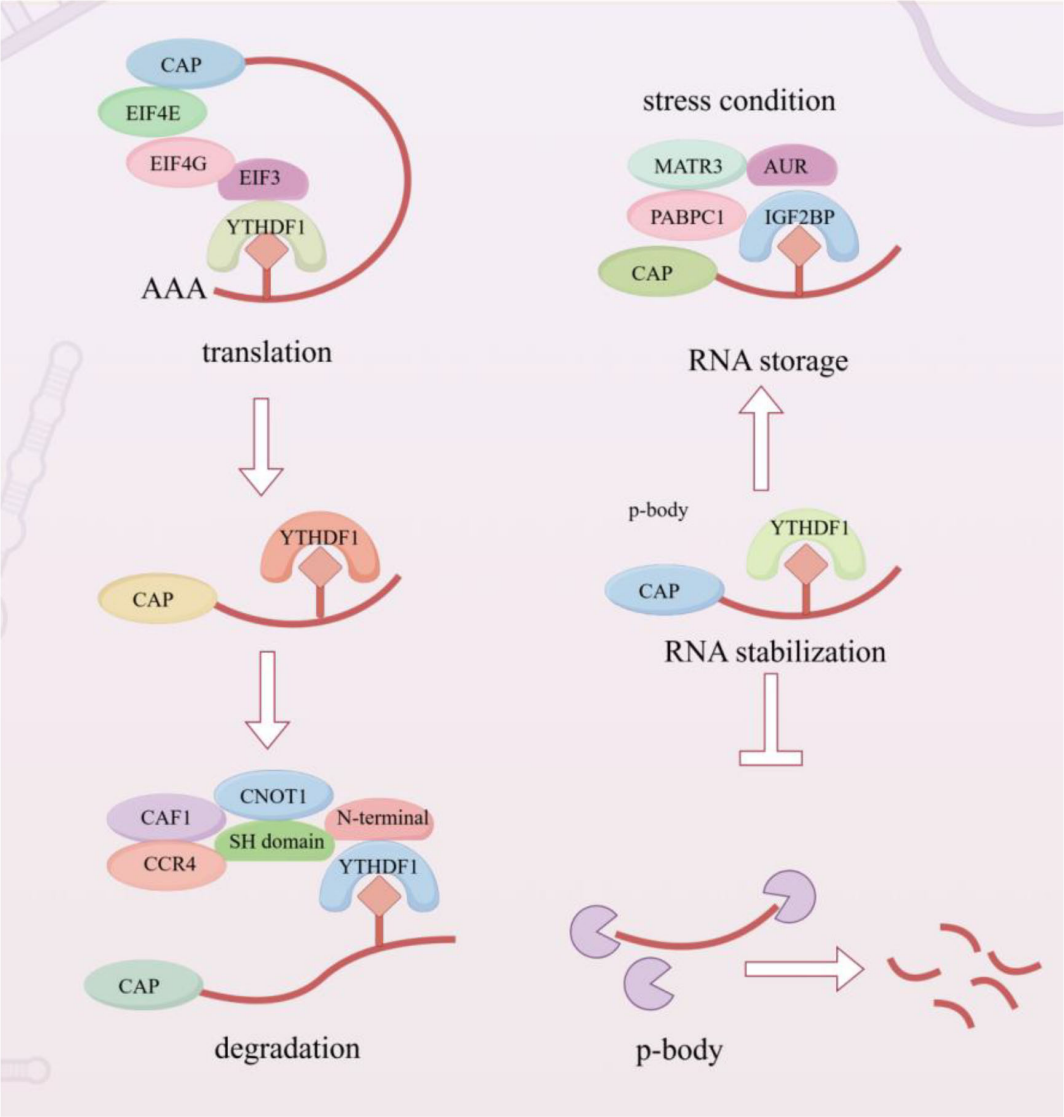
YTHDF1-mediated regulation of mRNA fate under stress conditions. This image summarizes the functional role of YTHDF1 as an m6A reader that directs methylated transcripts toward translation, storage, stabilization, or degradation depending on cellular context. Rather than emphasizing each associated factor individually, the image should be read as a process-oriented model of stress adaptation in NPC cells.

Reader proteins, particularly those containing YTH domains, interpret these modifications and thereby influence mRNA translation, stability, and stress-responsive RNA fate.The mechanism of mRNA fate regulation under stress conditions mediated by YTHDF1 is shown in **Figure 2**. In contrast, fewer proteins are known to read m^5^C, to date, only ALYREF has been confirmed to recognize m^5^C, primarily functioning in mRNA nuclear export (38).

The reversibility of m6A, mediated by enzymes such as FTO and ALKBH5, further emphasizes the plasticity of RNA-centered epigenetic regulation in cancer (39).

In NPC, RNA methylation is best understood as a dynamic post-transcriptional regulatory axis. By altering transcript stability and translational efficiency, it can influence stress adaptation, EMT, metastatic plasticity, and therapy response. It may also intersect with EBV-associated transcripts and non-coding RNA networks (40).

#### 2.1.3 Protein methylation: arginine/lysine methylation regulation in histones and non-histones

Protein methylation occurs in both histone and non-histone proteins and can influence transcriptional regulation, signaling activity, and cellular plasticity (41, 42). In general, lysine and arginine residues are the major targets of this modification, and their methylation is catalyzed mainly by lysine methyltransferases and protein arginine methyltransferases (43). These marks can be recognized by methylation-sensitive protein domains and can also be reversed by specific demethylases, making protein methylation a dynamic regulatory process rather than a static molecular event (44). In NPC, the importance of protein methylation lies primarily in its downstream regulatory consequences (42). By modulating chromatin state, transcriptional activity, and key oncogenic signaling pathways, protein methylation may influence invasion, metastatic maintenance, and treatment response (45, 46). Compared with DNA methylation, the evidence in NPC is relatively less systematized, but available studies suggest that aberrant methylation of histone and non-histone proteins may still contribute to aggressive tumor behavior through coordinated effects on gene expression and pathway activity (42). Accordingly, in this review, protein methylation is discussed mainly in terms of its disease relevance to NPC rather than as a comprehensive classification of methyltransferase and demethylase families.

### 2.2 Histone modifications relevant to NPC invasion and metastasis

Among the diverse histone-associated modifications, this review focuses only on those most relevant to NPC invasion, metastasis, and treatment adaptation, namely acetylation, phosphorylation, ubiquitination, and palmitoylation.

#### 2.2.1 Acetylation and deacetylation: roles in biological processes and protein function regulation​

Histone acetylation is a reversible modification mainly controlled by HATs and HDACs (46), with broad effects on chromatin accessibility and transcriptional activity (47, 48). Although acetylation participates in many physiological and pathological processes, its relevance in NPC is most evident in the regulation of EMT, proliferation, and treatment response (49). Accordingly, in this review, acetylation is considered primarily as a chromatin-based regulator of aggressive tumor phenotypes rather than as a general biochemical modification (50).

#### 2.2.2 Phosphorylation: a key modification in signal transduction

Phosphorylation is a central regulatory modification in signal transduction, and its functional impact depends on modification site, stoichiometry, and signaling context (51). In NPC, however, its importance is best understood through its contribution to invasive signaling networks, particularly those centered on AKT, β-catenin, STAT3, and other treatment-adaptive pathways (52).Therefore, the discussion here focuses on the role of phosphorylation in metastatic signaling rather than on its broader roles in general cell biology (53, 54).

#### 2.2.3 Ubiquitination: dual roles in protein degradation and functional regulation

Ubiquitination regulates protein stability, localization, and signaling output through coordinated action of E1, E2, and E3 enzymes (55). Beyond its classical role in proteasomal degradation, ubiquitination can also influence protein trafficking, complex formation, and functional activity in a context-dependent manner (56). These properties make it highly relevant to cancer biology, where altered ubiquitination contributes to aberrant signaling, adaptive stress responses, and malignant progression (56, 57).

In NPC, its importance is best understood in relation to the regulation of invasion-, metastasis-, and resistance-associated molecules, as changes in ubiquitination status may alter the abundance or activity of key effectors that sustain aggressive tumor behavior (58, 59). Therefore, the discussion here emphasizes its disease-specific significance in NPC rather than providing a broad overview of ubiquitination in general cell biology (59).

#### 2.2.4 Palmitoylation: lipid modification mediating protein localization and signal transduction

Palmitoylation is a reversible lipid modification that regulates membrane localization, trafficking behavior, and signaling dynamics by modulating the membrane affinity of target proteins (60, 61). This modification can alter the organization and activity of membrane-associated signaling complexes, thereby influencing key malignant phenotypes such as proliferation, migration, survival, and adaptive stress responses (62, 63).

Although evidence specifically linking palmitoylation to NPC remains relatively limited, its potential significance lies in the regulation of membrane-associated oncogenic pathways and treatment resistance, both of which are closely associated with tumor progression (64). Accordingly, in this review, palmitoylation is discussed as an emerging regulatory layer with possible relevance to NPC aggressiveness and therapeutic vulnerability, rather than as a broad overview of protein lipid modification in general biology (65).

## 3 Abnormal epigenetic regulation in nasopharyngeal carcinoma invasion and metastasis

Building on the mechanistic framework outlined above, the following section focuses on how these epigenetic layers become aberrantly organized in NPC and thereby contribute to invasion and metastasis.Cancer invasiveness is widely recognized as a multifactorial process driven by alterations in the tumor microenvironment, increased cellular motility, andEMT. Increasing evidence suggests that diverse epigenetic modifications promote NPC invasiveness through their regulation of these processes. Importantly, in NPC, these epigenetic abnormalities do not arise in isolation from viral biology; rather, EBV-associated epigenetic reprogramming should be regarded as a recurrent upstream process that helps drive broader epigenetic dysregulation during invasion and metastasis.

### 3.1 Aberrant methylation: a core event driving NPC invasion and metastasis

**Figure 3:**
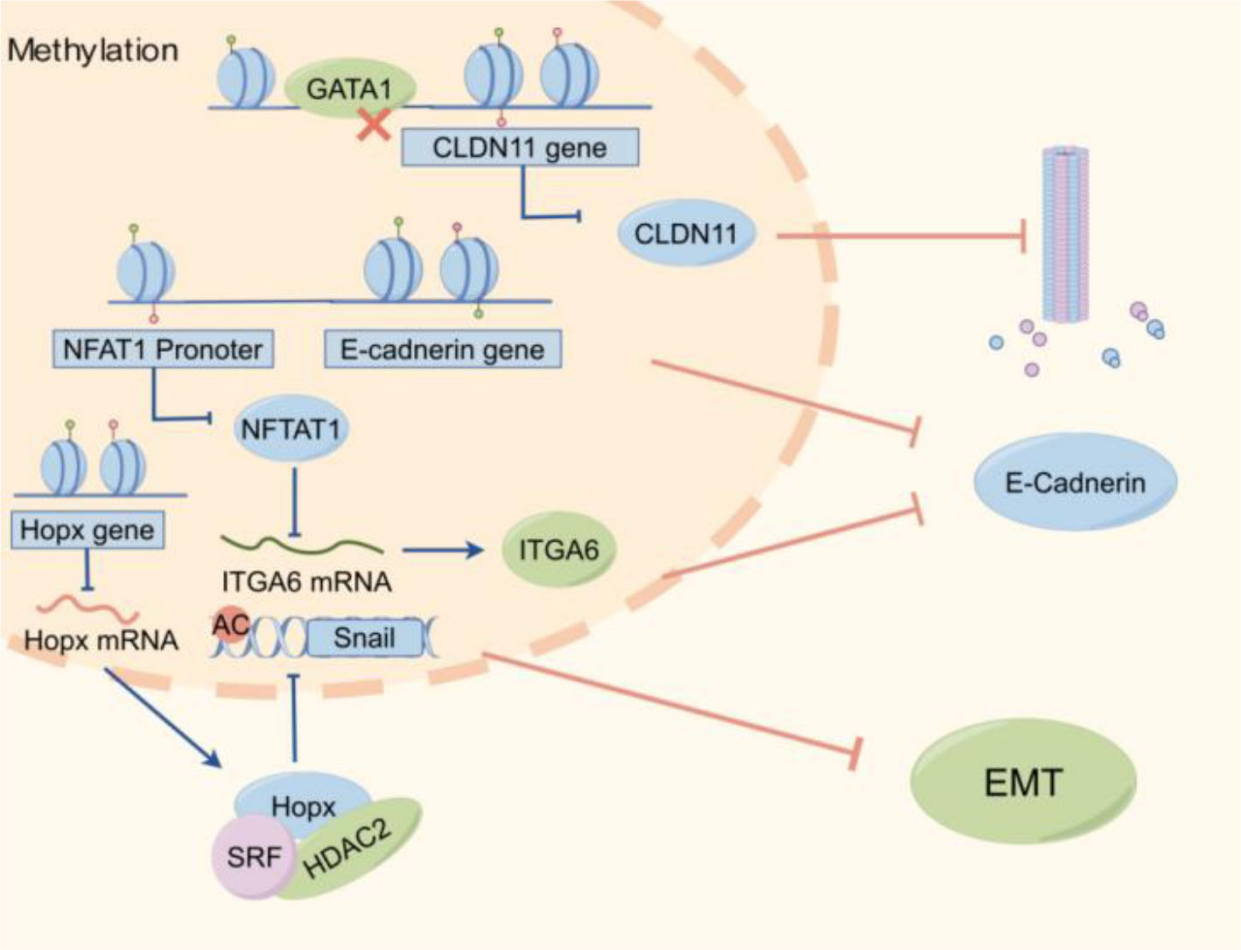
Regulatory network of aberrant methylation in NPC invasion and EMT. This image provides an integrated view of representative methylation-associated events that promote NPC aggressiveness. For clarity, it should be interpreted around three functional consequences: reduced cell-cell adhesion, induction of EMT, and enhancement of cytoskeletal remodeling or motility. The image is intended to illustrate convergence of multiple methylation changes on shared invasive phenotypes rather than to serve as an exhaustive catalog of all molecular interactions.

Aberrant methylation at multiple DNA loci and in proteins within cancerous epithelial cells has been linked to NPC invasiveness (66). In NPC, part of this aberrant methylation landscape is closely linked to EBV-associated host epigenetic reprogramming, a dynamic process that contributes to broader epigenetic dysregulation and the persistence of pro-metastatic phenotypesDNA methylation at distinct genomic loci and to varying extents can promote invasiveness through different molecular pathways. In this review, the mechanisms by which aberrant methylation enhances NPC invasiveness are broadly categorized into three major patterns, with representative examples summarized in **Table 1** and illustrated in **Figure 1**. First, hypermethylation of the E-cadherin promoter, a well-documented event listed in **Table 1**, suppresses E-cadherin expression, weakens intercellular adhesion, facilitates tumor cell detachment from the primary lesion, and thereby enhances the metastatic potential of NPC (78). Second, aberrant methylation of genes such as NFAT1 activates ITGA6-mediated signaling and inducesEMT (79).As shown in **Figure 1**, methylation of the NFAT1 promoter represses its expression and subsequently modulates ITGA6-associated pathways. Third, hypermethylation of CLDN11 promotes microtubule polymerization through regulation of TUBA1B and TUBB3, thereby enhancing NPC cell motility (75).This regulatory relationship is likewise depicted in **Figure 1** within the network linking methylated genes to EMT-related processes. Among these, abnormal methylation of E-cadherin and its promoter is the most common (**Table 1**) and is considered a potential target for cancer detection (80). These mechanisms are fully summarized in **Table 1**. The mechanisms by which aberrant methylation enhances NPC invasiveness can be broadly grouped into three recurring patterns, with representative examples summarized in **Table 1**. These include loss of cell–cell adhesion, activation of EMT-related signaling, and promotion of cytoskeletal or microenvironmental remodeling. As shown in **Table 1**, the convergence of multiple methylation-associated events on these shared invasive phenotypes highlights the central role of aberrant methylation in NPC invasion and metastasis.The regulatory network of key methylated genes/regions (e.g., CLDN11, NFAT1, E-cadherin, HOPX) and their roles in NPC invasiveness and EMT is visualized in **Figure 3**.

**Table 1:** Aberrant methylation, signaling pathways, and invasiveness mechanisms in nasopharyngeal carcinoma.

Aberrantly methylated genes or proteins	Signaling pathways or related proteins	Mechanisms of enhanced cell invasiveness	References
E-cadherin/E-cadherin promoter	E-cadherin	Tumor microenvironment alterations	(67)
TMEM130 promoter	TMEM130	Enhanced cell migration ability	(68)
miR-9-1	miR-9-1/HK2	Enhanced glycolysis, increased lactic acid secretion, and disruption of the microenvironment	(69)
14-3–3 sigma promoter	p53	Promoting lymph node metastasis and distant metastasis of NPC	(70)
PCDH8 promoter	PCDH8	Enhanced cell colony formation and migration ability	(71)
RERG	ERK/NF-κB	Enhanced tumor proliferation, migration, and angiogenesis ability	(72)
NFAT1	ITGA6	EMT and reduced E-cadherin levels	(73)
ZNF582	Nectin-3, NRXN3	Enhanced cell migration ability	(74)
CLDN11	TUBA1B, TUBB3	Microtubule polymerization	(75)
HOXA2	MMP-9	Tumor microenvironment alterations	(76)
HOPX	SNAIL	Therapy resistance triggered by chemotherapy and radiotherapy, promoting EMT	(77)

Taken together, the methylation-related literature shows a relatively consistent association between promoter hypermethylation of invasion-suppressive genes and the activation of EMT- or motility-related programs in NPC (81, 82). However, the strength of evidence is uneven across targets. For example, E-cadherin-associated methylation changes have been repeatedly linked to loss of cell-cell adhesion across multiple studies (83)., whereas several other methylation candidates remain supported mainly by single-cohort, single-model, or stage-restricted observations, making their generalizability less certain suggesting that methylation changes identified in primary tumors cannot always be directly extrapolated to metastatic behavior (78). These inconsistencies indicate that current evidence is stronger for association than for stage-specific causality, and that future studies should prioritize longitudinal sampling, metastatic lesion validation, and cross-cohort functional confirmation.

### 3.2 Aberrant phosphorylation: signal network remodeling and enhanced invasiveness

Evidence from multiple studies indicates that phosphorylation events involving AKT, β-catenin, and STAT3 are closely associated with NPC invasiveness. Signaling pathways including AKT/Wee1/CDK, EGFR/AKT/ERK, and JAK/STAT position these proteins as central hubs within the regulatory network governing NPC invasion (84–88). Accordingly, AKT, β-catenin, and STAT3 may serve as promising targets for therapeutic intervention. The major phosphorylation-associated signaling events implicated in NPC invasiveness are summarized in **Table 2**, which organizes the available evidence by phosphorylated proteins, related pathways, and downstream invasive consequences. As shown in **Table 2**, AKT-, β-catenin-, and STAT3-centered signaling axes recur across multiple studies, but the downstream phenotypes are not identical, ranging from EMT and immune microenvironment remodeling to cytoskeletal activation and therapy adaptation.

**Table 2:** Aberrant phosphorylation events, associated signaling pathways, and invasiveness mechanisms in nasopharyngeal carcinoma.

Aberrantly phosphorylated proteins	Signaling pathways or related proteins	Mechanisms of enhanced cell invasiveness	References
β-catenin	E-cadherin	Alterations in immune microenvironment	(84)
STAT3	RKIP/STAT3	EMT	(86)
GSK-3β	GSK-3β/Wnt/β-catenin	EMT	(88)
YAP	CircRILPL1/Hippo/YAP	Cell adhesion ability	(89)
EphA2	Shp2/Erk-1/2	Tumorigenicity	(90)
MLC2	CapG	Enhanced cell motility via increased actin assembly	(91)
p38	MKK3/p38	Lymph node metastatic ability	(92)
Src	Src	Upregulation of MMP2 activity and expression	(93)
Wee1, Akt, CDK1	Akt/Wee1/CDK1	Tumorigenicity	(94)
AKT	ARID1A	EMT	(95)

**Figure 4:**
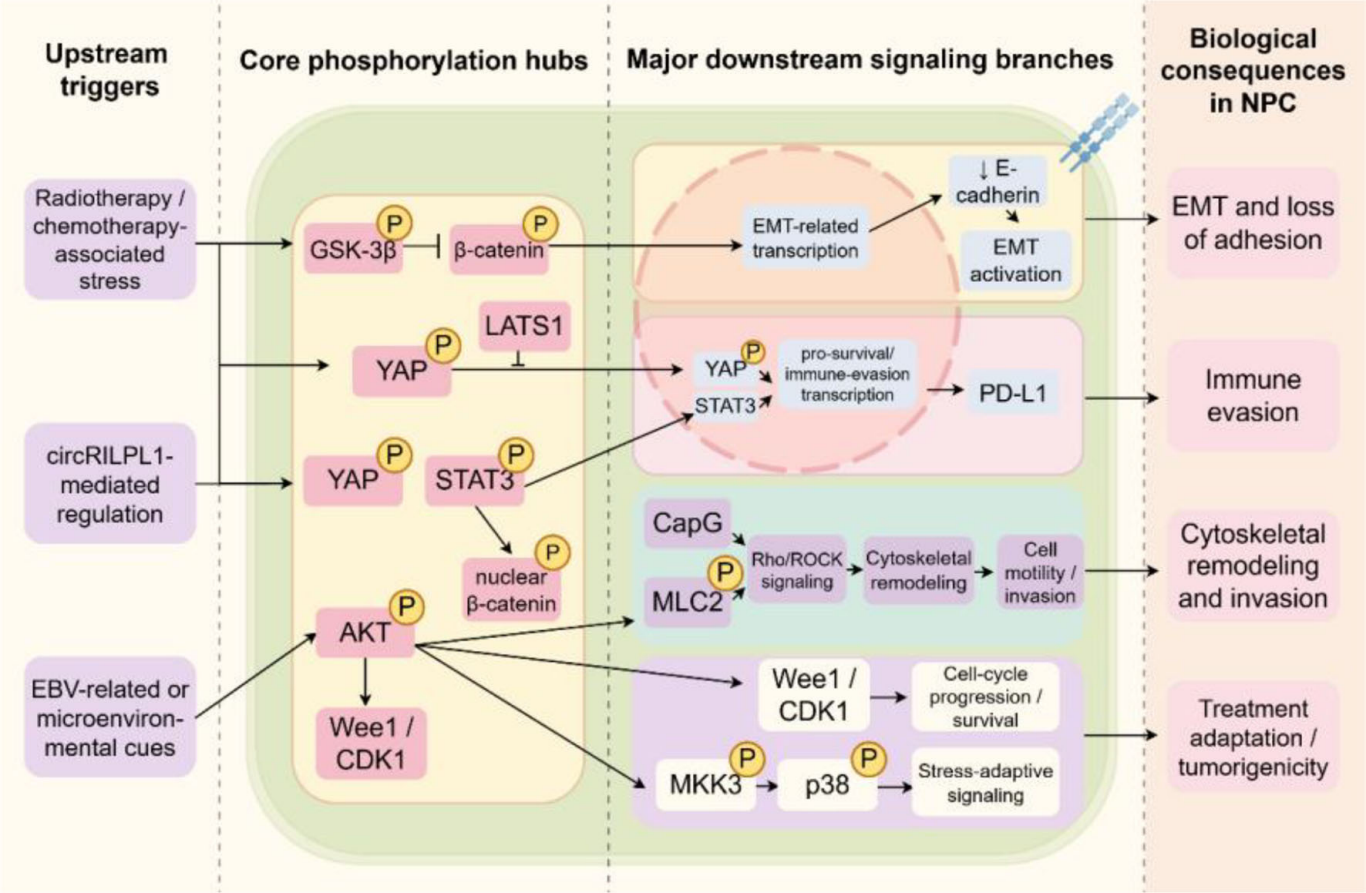
Phosphorylation-centered signaling network in NPC invasiveness and progression. This image presents a hierarchical overview of phosphorylation-associated signaling in NPC, linking upstream stimuli to core signaling hubs, downstream pathways, and biological outcomes. Upstream signals (therapy-related stress, circRNA regulation, and EBV or microenvironmental cues) converge on key hubs such as AKT, β-catenin, YAP, and STAT3. These hubs regulate distinct downstream branches controlling EMT, immune evasion (e.g., PD-L1), cytoskeletal remodeling, and stress-adaptive signaling. Together, these pathways drive major malignant phenotypes, including invasion, metastasis, and treatment adaptation. Only representative pathways are shown for clarity.

Although phosphorylation-related studies consistently implicate AKT, β-catenin, and STAT3 in NPC invasion, their roles are context-dependent and should not be regarded as interchangeable (89–93). In some settings, these pathways function as intrinsic drivers of aggressive behavior, whereas in others they appear to reflect therapy-induced adaptive reprogramming (96). Most available evidence remains preclinical, and clinically matched comparative studies are still limited (97). Therefore, these signaling axes are biologically compelling but not yet equally validated in terms of clinical relevance or reproducibility.The multi-step signaling network involving these molecules and their regulatory mechanisms is visualized in **Figure 4**.

### 3.3 Other abnormal epigenetic modifications: synergistic roles of palmitoylation, ubiquitination, and acetylation

**Figure 5:**
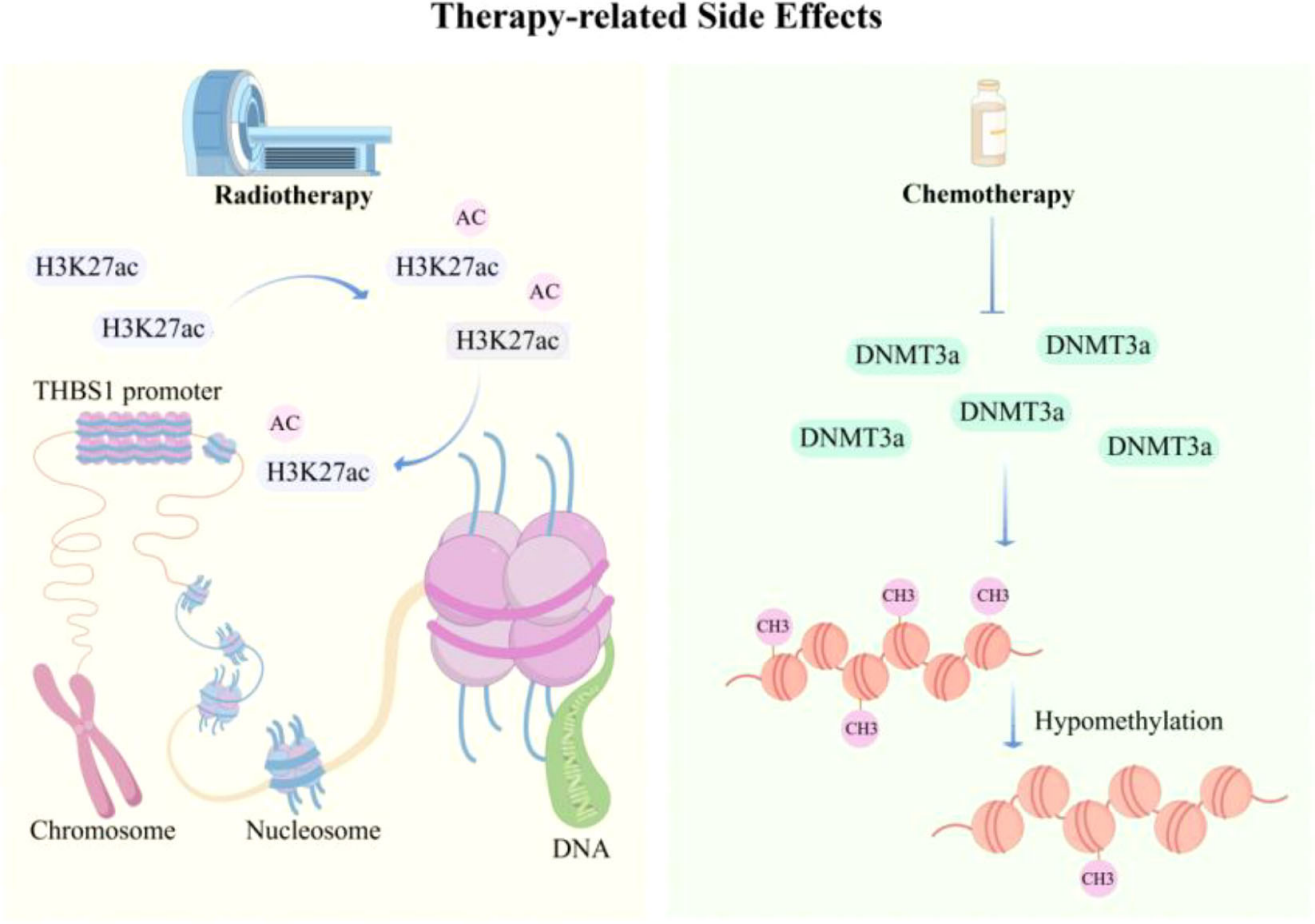
Therapy-induced epigenetic reprogramming associated with treatment-related adverse effects. This image highlights how radiotherapy- and chemotherapy-related stress may induce epigenetic reprogramming beyond tumor-intrinsic mechanisms, thereby contributing to clinically relevant adverse outcomes.

Beyond the extensively studied roles of methylation and phosphorylation, changes in NPC invasiveness have also been associated with other regulatory modifications, including ubiquitination and acetylation (98–100). Notably, in certain contexts, these epigenetic changes are not driven solely by intrinsic oncogenic events, but may also be induced by external stimuli arising from conventional treatments such as radiotherapy and chemotherapy. For instance, Bian et al. reported that radiotherapy reshapes the chromatin landscape in human dermal fibroblasts and epigenetically primes THBS1, leading to its aberrantly elevated expression during wound repair and ultimately impairing wound healing in cancer survivors (101). Liang et al. further showed that paclitaxel induces downregulation of DNMT3a in the medial prefrontal cortex (mPFC), accompanied by hypomethylation, thereby contributing to pain- and anxiety-like behaviors in male mice (102). Collectively, these findings underscore the need to more carefully re-evaluate the benefits and adverse consequences of conventional therapies, while also highlighting the clinical promise of more precise targeted interventions. The epigenetic mechanisms of treatment-related side effects caused by therapy are shown in **Figure 5**. Compared with DNA methylation- and phosphorylation-related mechanisms, the evidence supporting other epigenetic modifications in NPC invasiveness remains more fragmentary and is currently driven mainly by mechanistic plausibility and limited preclinical studies rather than by convergent multi-cohort validation.

**Figure 6:**
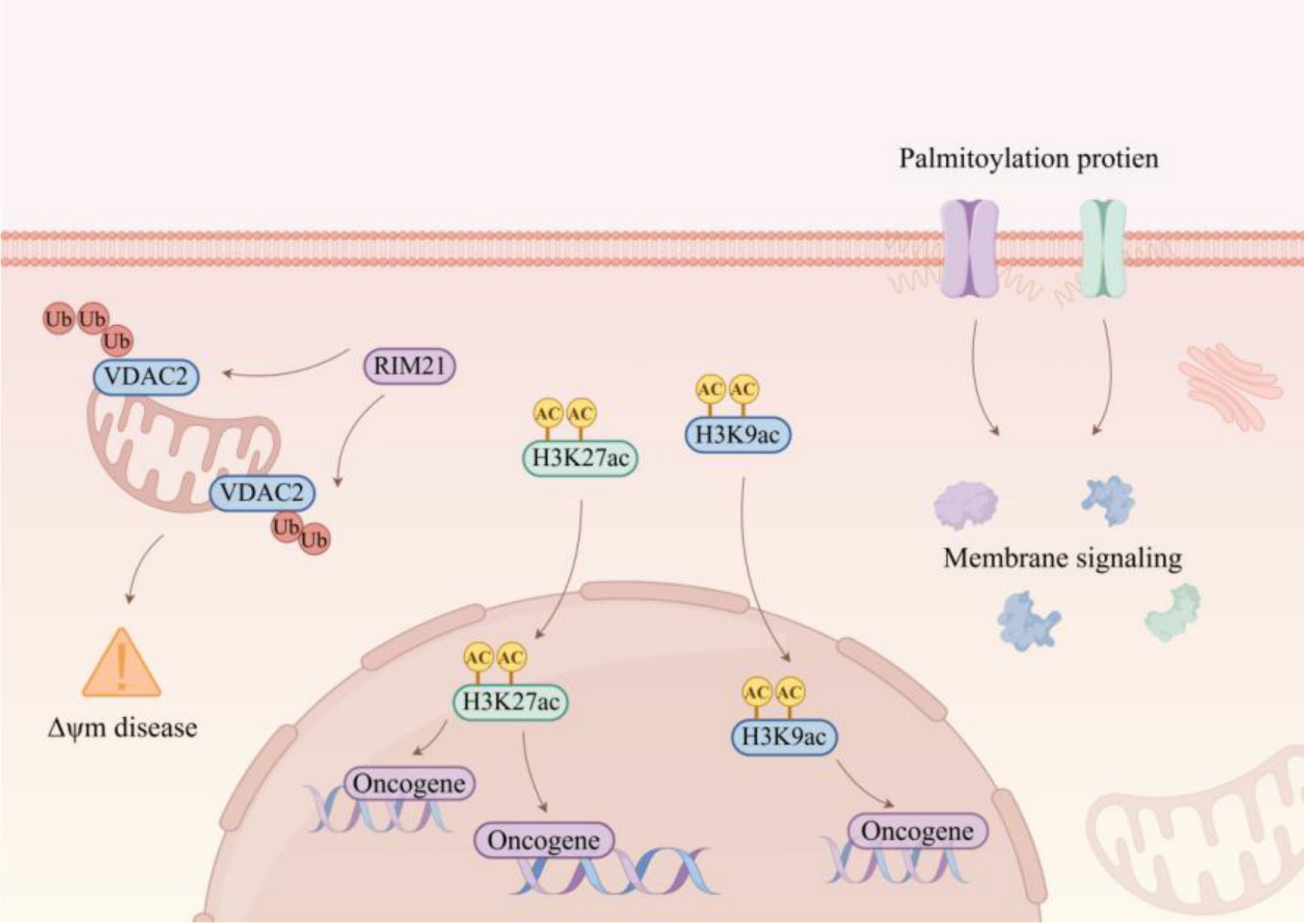
Crosstalk among palmitoylation, the RIM21–VDAC2 axis, and histone acetylation in NPC. This image is intended to highlight the mechanistic convergence of post-translational and chromatin-associated regulation on membrane signaling, mitochondrial function, and radioresistance. The emphasis is not on every individual interaction shown in the schematic, but on the central integrative role of the RIM21–VDAC2 axis within this regulatory context.

Given the close association between treatment resistance and aberrant histone-related modifications, increasing efforts have been directed toward the development of therapeutic strategies to overcome radioresistance and chemoresistance. For example, RIM21-mediated ubiquitination and degradation of VDAC2 impair mitochondrial function, thereby attenuating radioresistance in NPC (98). The schematic diagram of palmitoylation, RIM21-VDAC2 axis, and histone acetylation regulating membrane signaling and oncogene expression is shown in **Figure 6**.

### 3.4 Framework for evidence quality and translational maturity

Taken together, the evidence discussed above is not uniform in either strength or interpretability. Broadly, evidence can be considered relatively stronger when multiple independent studies converge on the same regulatory axis, when mechanistic perturbation is supported by *in vivo* validation, and when corresponding changes are observed in clinical NPC specimens (103). By this standard, promoter hypermethylation-associated silencing of invasion-suppressive genes and several phosphorylation-centered signaling nodes, such as AKT/β-catenin/STAT3-related pathways, are supported by comparatively stronger mechanistic evidence (104). In contrast, several emerging regulatory layers, particularly palmitoylation, selected ubiquitination events, and some non-coding RNA-associated pathways, remain supported mainly by single-model, single-cohort, or predominantly preclinical studies and should therefore be interpreted as preliminary rather than definitive. Importantly, much of the current NPC epigenetic literature still supports association more directly than causality (105). Although many studies demonstrate that epigenetic dysregulation correlates with EMT, invasion, metastasis, or adverse prognosis, fewer establish whether these changes are initiating drivers, context-dependent amplifiers, or secondary consequences of tumor progression or treatment exposure. This distinction is especially important in studies based primarily on expression correlation, endpoint tissue comparisons, or limited *in vitro* perturbation systems (106). Therefore, future work should place greater emphasis on longitudinal sampling, matched analyses of primary and metastatic lesions, causal perturbation designs, and cross-cohort validation in order to distinguish robust metastatic drivers from biologically plausible but still preliminary associations.To improve the consistency of evidence appraisal across sections, we applied a structured framework that considers both biological robustness and translational maturity (107). Briefly, evidence was interpreted across three broad levels: (i) hypothesis-generating evidence, mainly derived from *in vitro* studies, exploratory omics analyses, single-cohort correlative studies, or early mechanistic observations without independent validation (108); (ii) biologically supported preclinical evidence, defined by the presence of mechanistic perturbation data together with *in vivo* support and/or concordant findings in clinical NPC specimens (109); (iii) clinically actionable or clinically informative evidence, defined by biomarker use in practice, prospective clinical testing, or multi-cohort validation with clear translational relevance (110). In parallel, we used a semi-quantitative descriptive scale—preliminary, moderate, or relatively strong—to indicate overall evidence robustness according to convergence across independent studies, experimental depth, presence of *in vivo* validation, human specimen support, and degree of clinical confirmation (111). This framework was used to better distinguish promising mechanistic signals from findings that are already clinically informative or closer to implementation.

## 4 Value of epigenetic markers in prognosis of NPC invasion and metastasis

Beyond their mechanistic roles in tumor progression, epigenetic abnormalities also have potential clinical value as biomarkers for risk stratification, prognostic evaluation, and disease monitoring in NPC (112). This value is closely linked to their involvement in invasion and metastasis, which are major determinants of poor outcome (113). In addition, several epigenetic markers can be detected not only in tumor tissues but also in body fluids, supporting their translational relevance (114).

### 4.1 DNA methylation markers

DNA methylation markers have been extensively studied because they are linked to both NPC progression and metastatic prognosis (115). Several representative examples illustrate this dual role (116). Hypermethylation of CK1α promotes EMT through activation of Wnt/β-catenin signaling, whereas hypermethylation of UCHL1 and ACAT1 facilitates metastatic behavior by enhancing cytoskeletal remodeling and altering tumor metabolism, respectively (117, 118). In the setting of local invasion, hypermethylation of ECRG4 activates the AKT/GSK3β/β-catenin axis, and MFSD4A hypermethylation promotes EMT through PI3K–AKT–ERK signaling (119). In addition, EZH2–DNMT1-mediated hypermethylation of the miR-142-3p promoter relieves repression of ZEB2 and is associated with distant metastasis (120). Clinically, high DNMT3B expression and miR-142-3p methylation have both been associated with unfavorable survival outcomes (120, 121). Together, these findings support DNA methylation markers as biologically informative and clinically relevant candidates, although their broader translational applicability still requires standardized validation (122).

### 4.2 Non-coding RNA markers

Non-coding RNAs are particularly attractive prognostic candidates in NPC because they are dynamically involved in both invasion and metastasis and may also be assessed through liquid biopsy (123). Several lncRNAs and circRNAs, including LINC01503 (124), lnc-MRPL39-2, CircWHSC1, HOXA-AS2, and FOXD1-AS1, have been linked to EMT, metastatic spread, immune-related signaling, or glycolytic reprogramming (125), with some showing independent prognostic association (126). Beyond tissue-based assessment, circulating ncRNAs detected in serum or exosomes are of particular translational interest because they may enable non-invasive and real-time monitoring of progression-related risk (127, 128). Their clinical performance, however, remains sensitive to sample source, extraction efficiency, normalization strategy, and platform-related variation (129, 130).

### 4.3 Histone-related markers

The prognostic evidence is not equally mature across different classes of epigenetic markers (131). DNA methylation markers generally have stronger mechanistic support, but their clinical use is limited by tissue dependence and inter-lesional heterogeneity (132). By contrast, circulating ncRNAs offer clear advantages for dynamic monitoring, yet their reported performance is more vulnerable to pre-analytical variation and differences in normalization across studies (133). Histone-related markers remain promising but are currently supported by a comparatively smaller body of evidence (134). Overall, the available literature supports the potential of epigenetic biomarkers in NPC, but not yet their universal clinical readiness (135).

### 4.4 Comparative robustness and shared barriers

Despite their promise, epigenetic biomarkers still face several shared barriers to clinical translation (129, 136). Technical variability remains substantial, including differences in sample type, pre-analytical handling, extraction workflow, assay design, normalization strategy, and platform sensitivity (137, 138). Biological heterogeneity further complicates interpretation, because methylation and expression profiles may differ across primary tumors, recurrent lesions, and metastatic sites (131). These factors directly affect reproducibility and cross-cohort comparability (139). In addition, many proposed markers are still supported mainly by retrospective or single-center studies, highlighting the need for larger, prospectively standardized, multi-center validation (126, 140).

### 4.5 Clinical maturity and implementation perspective

Using the evidence framework outlined above, plasma EBV DNA can be regarded as clinically actionable, whereas selected methylation-based liquid biopsy candidates, such as plasma cfDNA VILL methylation, are better considered early clinically informative markers Most other proposed epigenetic biomarkers in NPC remain hypothesis-generating or early-validation findings rather than practice-ready tools (141). From an implementation perspective, plasma EBV DNA therefore remains the current benchmark for translational comparison (142, 143). The near-term role of emerging epigenetic biomarkers is likely to be complementary, rather than immediately substitutive, particularly in non-invasive detection, risk refinement, and multimodal monitoring strategies.Importantly, the translational relevance of these biomarkers extends beyond prognostic assessment, because some of the same epigenetic alterations may also help identify biologically actionable therapeutic targets. This connection provides a rationale for the transition to the therapeutic strategies discussed in the next section.

## 5 Novel therapeutic strategies for nasopharyngeal carcinoma based on epigenetics

### 5.1 Therapeutic strategies targeting abnormal DNA methylation

Although the therapeutic strategies discussed below are mechanistically promising, most of them remain at the preclinical or early translational stage. In NPC, the available evidence is still dominated by cell-line experiments, xenograft models, and limited tissue-based observations, whereas prospective clinical validation, real-world feasibility, and biomarker-guided patient selection remain insufficiently developed. Accordingly, therapeutic enthusiasm should be interpreted in light of evidence maturity, because mechanistic targetability alone does not yet establish clinical tractability, safety, or durable efficacy in biomarker-defined NPC populations (144).

**Figure 7:**
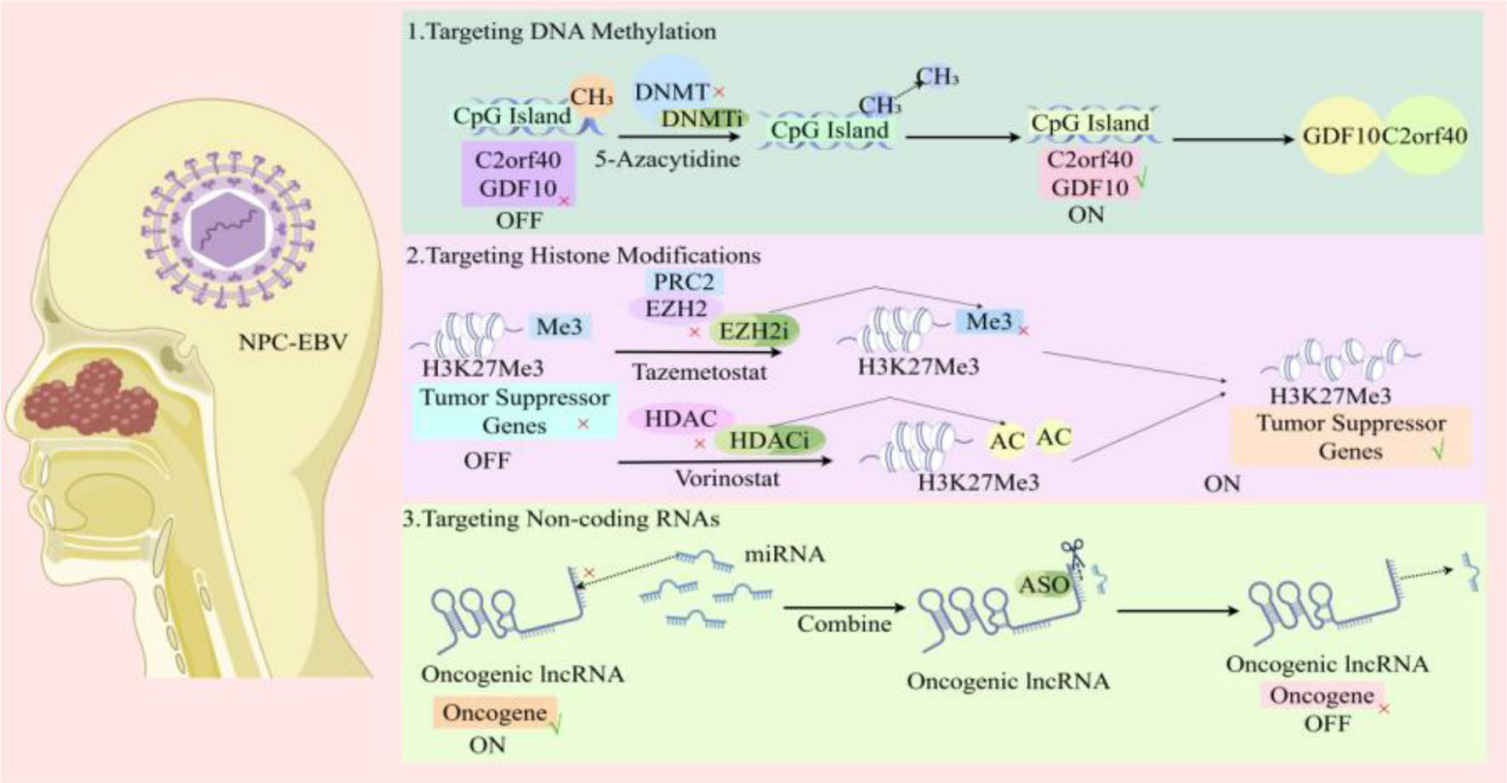
Major epigenetic therapeutic strategies in NPC. This image summarizes three principal intervention directions discussed in this review, targeting DNA methylation, histone modifications, and non-coding RNA regulation. It is designed as a conceptual overview of therapeutic logic, linking epigenetic targets to their expected antitumor consequences in invasion, metastasis, and treatment response.

In the invasive progression of NPC, targeting aberrant DNA methylation may provide a precise strategy to suppress tumor invasion. First, direct inhibition of epigenetic regulatory enzymes can impair invasive capacity. Silencing DNMT3B markedly reduces the local infiltrative potential of tumor cells by suppressing EMT, as reflected by increased E-cadherin expression and decreased N-cadherin expression (121). Second, demethylation of key hypermethylated genes can interrupt invasion-associated signaling. C2orf40 is frequently hypermethylated in NPC, and restoration of its expression through demethylation inhibits transmatrix migration by suppressing PI3K/AKT-mediated cytoskeletal reorganization, including reduced F-actin polymerization (145). Likewise, demethylation of ARNTL reverses CDK5-driven pseudopodia formation, thereby attenuating the invasive activity at the leading edge of tumor cells (146). Finally, interventions targeting methylation-associated metabolic reprogramming may weaken the pro-invasive microenvironment. SLC27A6 is hypermethylated and downregulated in NPC, whereas its re-expression following demethylation promotes lipid accumulation and suppresses integrin cluster activation as well as local infiltration through regulation of cholesterol membrane distribution (147, 148). A schematic diagram of epigenetic therapeutic strategies for NPC is shown in **Figure 7**.

Representative DNA hypermethylation targets involved in NPC invasion and metastasis are summarized in **Table 3**, together with their proposed mechanisms, experimental models, and associated signaling pathways. Importantly, **Table 3** shows that although several targets have strong mechanistic plausibility, the supporting evidence remains dominated by cell-line and xenograft studies, with limited prospective or clinically intervention-oriented validation.Most studies rely on NPC cell lines and xenograft models, whereas clinical validation is limited and generally restricted to correlative tissue observations rather than prospective therapeutic testing (151). Therefore, the current evidence more strongly supports biological plausibility than immediate real-world applicability.

**Table 3:** DNA hypermethylation targets, mechanisms, and experimental evidence in nasopharyngeal carcinoma invasion and metastasis.

Epigenetic modification	Mechanism	Experimental models	Pathway	References
DNMT3B upregulation	Radiation → ↑DNMT3B → hypermethylation of p53/p21 → ↓apoptosis + ↓cell cycle arrest → radioresistance; DNMT3B silencing → ↓EMT → ↓migration/invasion → ↑radiosensitivity.	CNE-2/SUNE-1 cells NPC xenograft mouse model	-	(121)
miR-142-3p promoter hypermethylation	EZH2 → DNMT1 → miR-142-3p ↓ → ZEB2 ↑ → EMT ↑ → Metastasis	NPC cell lines, Clinical tissues	EZH2/DNMT1 → miR-142-3p/ZEB2 → EMT	(120)
NFAT1 promoter hypermethylation	NFAT1 ↓ → ITGA6 ↑ → EMT ↑ → Metastasis	NPC cell lines, NPC xenograft mouse model/NPC tissues	NFAT1/ITGA6 → EMT	(73)
C2orf40 hypermethylation	C2orf40 overexpression → ↓PI3K/AKT/mTOR → ↓CCNE1/CDK1 → G2/M arrest; ↑apoptosis + ↓DNA repair (↓BRCA1/RAD51) → ↓migration/invasion + ↑chemo/radiosensitivity.	5-8F/HONE1 cells:	PI3K/AKT/mTOR → CCNE1/CDK1 → Cell cycle; BRCA1/RAD51 → DNA repair	(145)
SLC27A6 hypermethylation	SLC27A6 downregulation → ↓lipid uptake → ↓TG/T-CHO → ↓CSC markers (CD24/CD44); SLC27A6 re-expression → ↑EMT + ↑lipid droplets → ↑migration/invasion + ↑CSC maintenance → pro-metastatic.	S18/S26 cells: Transwell NPC xenograft mouse model	Lipid metabolism → CSC markers (CD24/CD44) → EMT	(147)
GDF10 promoter hypermethylation	GDF10 ↓ → TGF-β/Smad ↓ + NF-κB ↑ → Proliferation ↑/EMT ↑ → Metastasis	NPC cell lines, NPC xenograft mouse model	TGF-β/Smad; NF-κB → Proliferation/EMT	(149)
SEPT9_v2 promoter hypermethylation	SEPT9_v2 ↓ → miR-92b-3p ↓ → FZD10 ↑ → Wnt/β-catenin ↑ → Migration ↑/Invasion ↑	NPC cell lines, NPC tissues	SEPT9_v2/miR-92b-3p/FZD10 → Wnt/β-catenin	(137)
SHISA3 promoter hypermethylation	SHISA3 ↓ → TRIM21 ↑ → SGSM1 ↓ → MAPK ↑ → Metastasis ↑	NPC cell lines, NPC xenograft mouse model	SHISA3/TRIM21/SGSM1 → MAPK	(150)

↑: significant increase in the level/expression of the corresponding factor; ↓: significant decrease in the level/expression of the corresponding factor; →: indicates a causal or sequential relationship between two related factors in the proposed mechanism.

Despite these promising therapeutic prospects, current strategies remain subject to several important limitations. A major challenge lies in the functional duality of certain targets. For instance, although SLC27A6 demethylation suppresses tumor proliferation, it may also enhance cancer stem cell (CSC) traits through lipid metabolic reprogramming, thereby potentially promoting local infiltration (147). Second, resistance-associated feedback loops are difficult to interrupt. Radiotherapy can induce upregulation of DNMT3B, resulting in remethylation of target genes such as p21 and consequently reducing the durability of therapeutic responses (121, 152). Third, the mechanisms underlying chemoresistance are highly complex. Methylation-mediated silencing of ARNTL confers cisplatin resistance through CDK5 activation, yet the downstream effectors of CDK5 remain insufficiently targeted (146). In parallel, compensatory activation of the PI3K pathway in tumors with low C2orf40 expression may limit the efficacy of single-agent demethylation therapy (145).

To overcome these limitations, anti-invasive therapeutic strategies for NPC could be further optimized in three directions. For targets with context-dependent or contradictory effects, pathway-based combination strategies may provide a more effective solution. For example, SLC27A6 demethylation therapy could be combined with lipid metabolism inhibitors, such as DGAT1 inhibitors, to block lipid droplet formation while simultaneously suppressing CSC-associated properties. For resistance-associated feedback circuits, cascade inhibition may be required. One potential approach would be the development of DNMT3B siRNA-based nanodelivery systems administered concurrently with radiotherapy to prevent radiation-induced DNMT3B upregulation and subsequent remethylation. In ARNTL-silenced tumors, combining CDK5 inhibitors, such as Roscovitine, with demethylating agents may improve cisplatin sensitivity. In addition, dynamic methylation-guided stratification may help identify patients most likely to benefit from specific interventions, such as selecting candidates for PI3K inhibitors on the basis of C2orf40 methylation status or using DNMT3B methylation patterns to optimize the timing of radiosensitization.In the metastatic progression of NPC, targeting aberrant DNA methylation may disrupt the metastatic cascade at multiple levels. First, restoration of methylation-silenced miRNAs can directly suppress metastatic initiation. Demethylation of miR-142-3p inhibitsEMT and circulating tumor cell formation through targeted degradation of ZEB2 mRNA (120). Second, re-expression of hypermethylation-silenced tumor suppressor genes can interfere with the microenvironment required for metastatic colonization. GDF10 is frequently hypermethylated in NPC, and its reactivation through demethylation suppresses angiogenesis and extracellular matrix remodeling in metastatic lesions by inhibiting nuclear accumulation of Smad2 and NF-κB (149).Similarly, SEPT9_v2, a variant of SEPT9, is hypermethylated and downregulated in NPC, whereas its re-expression inactivates Wnt signaling through the miR-92b-3p/FZD10 axis and thereby suppresses clonal outgrowth at distant sites (137). Finally, targeting the methylation of metastasis-promoting genes may weaken migratory and adhesive capacity. Demethylation of SHISA3 enhances SGSM1 stability and inhibits MMP-mediated tissue invasion (150), whereas demethylation of NFAT1 suppresses endothelial adhesion of metastatic cells by downregulating ITGA6 transcription (73).

However, current therapeutic approaches to metastatic disease still face three major obstacles. The first is the heterogeneity of metastatic lesions. For example, the methylation status of SEPT9_v2 may differ between primary and metastatic tumors, meaning that single-site sampling may fail to detect micrometastatic disease (137). The second is inefficient drug delivery. Therapeutic agents such as miR-142-3p mimics or GDF10 expression vectors often have limited ability to cross the blood–brain or blood–bone barriers and therefore may not adequately reach metastatic lesions (120, 149, 153). The third is compensatory pathway activation. Although re-expression of NFAT1 inhibits ITGA6, metastatic cells may bypass this effect through compensatory activation of integrin αVβ3 (73). Likewise, demethylation of SHISA3 appears to be ineffective in established bone metastases, possibly because these lesions sustain survival and colonization through PI3K/AKT signaling independently of SHISA3 (154).

Addressing these limitations will require innovative technical approaches. One promising direction is the development of dynamic multi-gene methylation monitoring, for example by integrating SEPT9_v2, miR-142-3p, and NFAT1 into a methylation panel for ctDNA-based detection of micrometastases and for guiding the timing of intervention. A second direction is the engineering of intelligent delivery systems, such as pH-responsive liposomes carrying GDF10 plasmids to target acidic metastatic niches, or exosome-based platforms for delivering miR-142-3p mimics across the blood–brain barrier. A third direction is compensatory pathway co-targeting. For instance, NFAT1 demethylation therapy could be combined with integrin αVβ3 inhibitors such as Cilengitide, whereas SHISA3-hypermethylated bone metastases may require the addition of RANKL inhibitors such as Denosumab to suppress osteolytic destruction.

### 5.2 Intervention approaches targeting abnormal histone modifications

**Figure 8:**
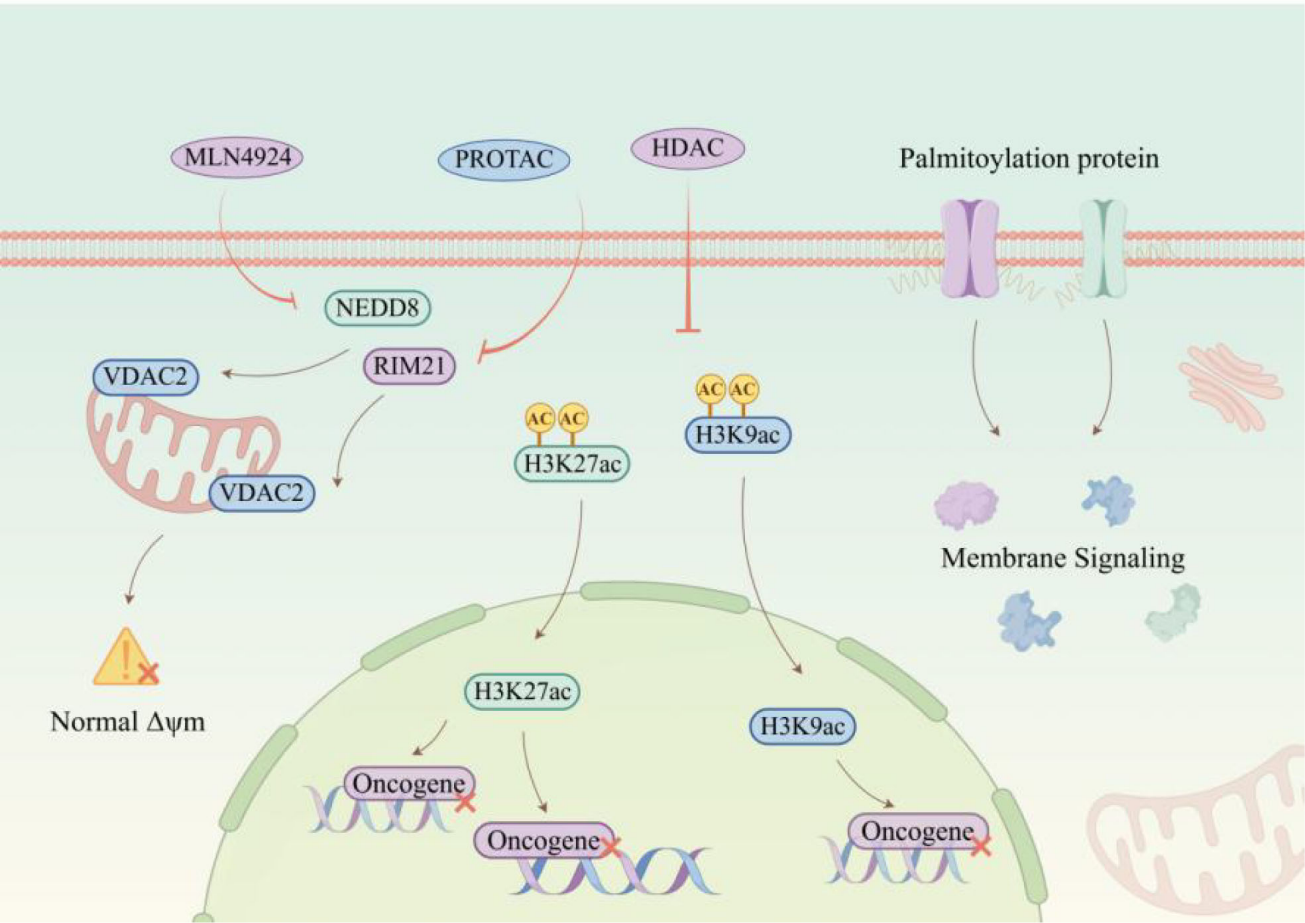
Epigenetic therapy strategies targeting HDAC, palmitoylation, and RIM21-VDAC2 axis. This image outlines targeted therapeutic interventions for NPC, including PROTAC-mediated HDAC inhibition and MLN4924 (a NEDD8-activating enzyme inhibitor). These agents modulate palmitoylated proteins, RIM21, and VDAC2, restoring the balance of histone acetylation (H3K27ac and H3K9ac) and mitochondrial membrane potential (Δψm). By inhibiting aberrant membrane signaling pathways and oncogene activation, this combinatorial strategy suppresses NPC progression and overcomes therapy resistance.

The invasive phenotype of NPC is closely linked to aberrant histone modifications. Studies have shown that the histone demethylase KDM4A alleviates transcriptional repression of HIF1α by reducing H3K9me3 levels, thereby activating the downstream DDIT4/mTOR signaling axis and promoting tumor cell migration and invasion (155). In addition, HDAC7 upregulates EphA2 by suppressing miR-4465 expression, which in turn inducesEMT and enhances invasive capacity.^102 Targeting these mechanisms offers potential therapeutic opportunities. Small-molecule KDM4A inhibitors, such as JIB-04, can restore H3K9me3 levels and block mTOR signaling (155), whereas HDAC7-selective inhibitors, such as TMP195, or miR-4465 mimics may reverse EphA2 overexpression and suppress the invasive phenotype (156). Collectively, these strategies inhibit tumor invasion by interfering with histone modifier activity and reshaping chromatin accessibility.NPC metastasis is likewise governed by multidimensional epigenetic mechanisms. CBX1 suppresses the tumor suppressor MAP7 through H3K9me3-mediated heterochromatin formation, while simultaneously activating STAT1 signaling to upregulate PD-L1, thereby promoting immune evasion and distant metastasis (157). HDAC4 also represses E-cadherin promoter activity through deacetylation, inducing EMT and driving lung metastasis (158). Therapeutically, these findings suggest that restoration of the chromatin state of key genes, or interference with associated non-coding RNA regulatory networks, may effectively suppress metastatic progression. Epigenetic therapeutic strategies targeting HDACs, palmitoylation, and the TRIM21–VDAC2 axis are summarized in **Figure 8**, whereas the principal histone-related effectors relevant to invasion and metastasis are compared in **Table 4**. As shown in **Table 4**, the currently emphasized histone-associated mechanisms in NPC converge mainly on chromatin-mediated control of EMT, invasive signaling, and immune-related metastatic regulation, but the number of well-characterized effectors remains relatively limited.

**Table 4:** Histone modifications effectors, mechanisms, and associated pathways in nasopharyngeal carcinoma invasion and metastasis.

Epigenetic markers	Mechanism	Experimental models	Pathway	Reference
Histone demethylase KDM4A	KDM4A ↑ → H3K9me3 ↓ at HIF1α promoter → HIF1α ↑ → DDIT4 ↑ → mTOR pathway activation → Promotes migration/invasion	SUNE1 cells, NPC xenograft mouse model	KDM4A/H3K9me3 → HIF1α/DDIT4 → mTOR pathway	(155)
Histone deacetylase HDAC7	HDAC7 ↑ → miR-4465 ↓ → EphA2 ↑ (miR-4465 target) → Promotes proliferation, migration, and invasion	NPC cell lines, NPC xenograft mouse model	HDAC7/miR-4465 → EphA2	(156)
Histone methylation reader CBX1	CBX1↑ → H3K9me3-mediated heterochromatin↑ → MAP7↓ → Migration/Invasion↑ CBX1↑ → STAT1 activation → PD-L1↑ → Immune evasion	NPC cell lines Metastatic NPC tissues	CBX1/H3K9me3 → MAP7; CBX1/STAT1 → PD-L1	(157)
Histone deacetylase HDAC4	HDAC4↑ → E-cadherin promoter deacetylation → E-cadherin↓ → EMT↑ (N-cad↑, Vimentin↑) HDAC4↑ → G1/S transition↑ → Proliferation↑	NPC cell lines, NPC xenograft mouse model/NPC tissues	HDAC4 → E-cadherin → EMT; HDAC4 → G1/S transition	(158)

↑: significant increase in the level/expression of the corresponding factor; ↓: significant decrease in the level/expression of the corresponding factor; →: indicates a causal or sequential relationship between two related factors in the proposed mechanism.

Despite the therapeutic promise of epigenetic targeting in suppressing NPC invasion and metastasis, its clinical translation still faces several major obstacles. Strategies aimed at inhibiting invasion are limited by two central challenges. First, although KDM4A inhibitors such as JIB-04 can suppress mTOR signaling by restoring H3K9me3 levels, H3K9me3 is a broad histone modifications mark involved in global chromatin regulation. Its manipulation may therefore disrupt chromatin stability in normal cells and lead to systemic toxicities, including myelosuppression (155). Second, although HDAC7 inhibitors or miR-4465 mimics may target the EphA2 axis, miR-4465 itself has pleiotropic regulatory functions and may unintentionally activate pro-survival pathways such as PI3K/AKT, thereby promoting adaptive resistance rather than durable tumor suppression (156). The limitations of metastasis-directed strategies are even more complex. On the one hand, CBX1 promotes metastasis through a dual epigenetic–immune mechanism involving MAP7 repression and PD-L1 upregulation, yet targeting CBX1 alone may be undermined by compensatory enhancement of STAT1 signaling (157). On the other hand, although the HDAC4 inhibitor Tasquinimod can inhibit EMT, its ability to epigenetically reactivate E-cadherin is constrained by the hypoxic tumor microenvironment, resulting in heterogeneous therapeutic responses (158). In addition, siRNA therapy targeting lncRNA FAM225A can block the cGAS–STING pathway, but its *in vivo* delivery efficiency remains low and it may also provoke excessive immune activation (159). Moreover, the dynamic interaction of the EBF3/EGR1 complex further increases the technical difficulty of achieving effective multi-target synergistic intervention (132). Collectively, these limitations reflect the intrinsic complexity and compensatory capacity of epigenetic regulatory networks, indicating that single-target approaches are unlikely to achieve sustained long-term efficacy.To overcome these bottlenecks, therapeutic strategies should be optimized with respect to precision, synergy, and delivery efficiency. First, at the level of precision, spatiotemporally restricted epigenetic editing tools may offer substantial advantages. For example, CRISPR/dCas9-mediated epigenetic editing, such as fusion of dCas9 to the TET1 catalytic domain, enables locus-specific removal of DNA methylation marks at promoters including PD-L1, NFAT1, and SEPT9_v2. This approach avoids the systemic toxicity associated with global demethylation and provides improved spatial and temporal control over epigenetic modulation. It may also complement tumor-selective strategies designed to reshape H3K9me3 and histone acetylation states in malignant cells through targeted regulation of KDM4A- and HDAC7-associated pathways (155, 156). Second, to address compensatory signaling, multi-pathway co-targeting regimens should be developed. In the case of CBX1, combining m6A modification inhibitors, to reduce CBX1 expression, with STAT1 antagonists such as Fludarabine may simultaneously suppress MAP7 silencing and PD-L1 upregulation, thereby limiting immune evasion (157). Further innovation in delivery systems will also be essential. For instance, pH-responsive nanoparticles carrying HDAC4 inhibitors together with hypoxia-activated E-cadherin enhancers could improve local drug accumulation within metastatic niches and enhance therapeutic specificity (158). For lncRNA-targeted interventions, exosome-based delivery systems capable of co-transporting FAM225A siRNA and negative regulators of the STING pathway, such as A151, may both silence prometastatic RNA signals and reduce the risk of cytokine storm (159). Finally, integration of single-cell epigenomics with artificial intelligence-based predictive models may enable dynamic monitoring of chromatin-state changes in drug-resistant clones, thereby providing a framework for real-time adjustment of treatment strategies (132). Together, these advances could shift epigenetic therapy for NPC from single-target intervention toward multidimensional regulation, ultimately improving both the precision and durability of treatment. Taken together, these limitations indicate that, despite strong mechanistic rationale, histone-targeting strategies in NPC have not yet achieved the level of clinical validation required for routine application, particularly with respect to safety, durable efficacy, and implementation in biomarker-defined patient populations.

### 5.3 Non-coding RNA-targeted therapy: precision intervention based on epigenetic networks

The local invasive capacity of NPC is closely associated with epigenetic dysregulation of long non-coding RNAs (lncRNAs). For example, DRAIC promotes cytoskeletal remodeling and invasive phenotypes by sponging miR-122 and thereby upregulating SATB1 expression (160). Aberrant regulation of microRNAs (miRNAs) also contributes to the invasive process; reduced miR-873 expression accelerates tumor infiltration through activation of the AKT signaling pathway (161). On the basis of these mechanisms, RNA-targeted therapeutic strategies may intervene in two major ways: first, by using antisense oligonucleotides (ASOs) to silence pro-invasive lncRNAs, such as FAM225A and DRAIC; and second, by delivering miRNA mimics, such as miR-873 mimics, to restore tumor-suppressive activity. Studies have shown that such approaches can significantly inhibit tumor cell migration and matrix invasion (160, 162), highlighting their potential to reverse the invasive phenotype through precise modulation of RNA interaction networks.The molecular basis of distant NPC metastasis involves not only functional loss of miRNAs and lncRNAs, but also their direct epigenetic silencing. Specifically, EZH2 and DNMT1 cooperatively induce H3K27me3 enrichment and DNA hypermethylation at the miR-142-3p promoter, thereby relieving repression of ZEB2 and promotingEMT. At the same time, reduced expression of the tumor-suppressive lncRNA EP300-AS1 impairs activation of the TFAP2C/CST6 axis, leading to inactivation of metastasis-suppressive pathways (163). Notably, super-enhancer-derived RNAs (seRNAs) can accelerate lymph node metastasis by stabilizing the hnRNPK/MICAL2 complex and thereby promoting cytoskeletal remodeling (164). Targeting these mechanisms, combinatorial strategies integrating epigenetic agents with RNA-based therapies appear particularly promising. For instance, EZH2 inhibitors, such as Tazemetostat, or DNMT1 inhibitors, such as decitabine, may restore miR-142-3p expression (120), whereas exogenous supplementation of EP300-AS1 or selective degradation of seRNAs may interrupt the metastatic cascade (163, 164). Taken together, re-establishing the balance between epigenetic regulation and transcriptomic output may represent an important strategy for suppressing NPC metastasis Key non-coding RNA regulators involved in NPC invasion and metastasis are summarized in **Table 5**, which integrates epigenetic modification type, mechanistic consequence, experimental support, and pathway context. As highlighted in **Table 5**, non-coding RNA dysregulation in NPC operates through diverse mechanisms—including promoter hypermethylation, m6A-associated stabilization, and ceRNA-like signaling—but most candidate therapeutic approaches remain constrained by delivery, stability, and validation challenges.

**Table 5:** Non-coding RNA regulators, mechanisms, and associated pathways in nasopharyngeal carcinoma invasion and metastasis.

Epigenetic modification types	Mechanism	Experimental models	Pathway	References
miR-142-3p promoter hypermethylation	EZH2-recruited DNMT1 → miR-142 locus hypermethylation → miR-142-3p↓ → ZEB2↑ → EMT↑ → Migration/Invasion↑ → Metastasis↑	*In vitro*: NPC cell lines (5-8F, 6-10B, CNE2) *In vivo*: NPC xenograft mouse model/NPC tissues	EZH2/DNMT1 → miR-142-3p/ZEB2 → EMT	(120)
lncRNA FAM225A	m^6^A modification → ↑FAM225A stability → sponges miR-590-3p/miR-1275 → ↑ITGB3 → activates FAK/PI3K/Akt signaling → ↑proliferation/↑invasion/↑metastasis	*In vitro*: NPC cell lines; *In vivo*: NPC xenograft mouse model	m^6^A/FAM225A → miR-590-3p/miR-1275/ITGB3 → FAK/PI3K/Akt	(162)
lncRNA DRAIC	DRAIC → sponges miR-122 → ↑SATB1 → ↑proliferation/↑migration/↑invasion → Promotes NPC progression	*In vitro*: NPC cell lines; *In vivo*: Not specified	DRAIC/miR-122 → SATB1	(160)
miR-873 downregulation	↓miR-873 → ↑ZIC2 → activates AKT signaling → ↑stemness/↑tumorigenicity → Promotes CSC-mediated invasion/metastasis	*In vitro*: CD133^+^ NPC stem cells/sphere formation *In vivo*: Tumorigenicity assay	miR-873/ZIC2 → AKT	(161)
lncRNA EP300-AS1 downregulation	EP300-AS1↓ → TFAP2C binding↓ → CST6↓ → EMT↑ (E-cadherin↓, N-cadherin↑) → Migration/Invasion↑ → Metastasis↑	*In vitro*: NPC cell lines (CNE1, CNE2, HONE1) *In vivo*: NPC xenograft mouse model/NPC clinical samples	EP300-AS1/TFAP2C → CST6 → EMT	(163)
lncRNA LINC00173 upregulation	LINC00173↑ → Binds RAB1B → PA2G4/SDF4 secretion↑ → Tumor growth↑ → Lymph node/Lung metastasis↑	*In vitro*: NPC cell lines (C666-1, SUNE1, HNE1) *In vivo*: NPC xenograft mouse model/NPC tissues	LINC00173/RAB1B → PA2G4/SDF4	(164)

↑: significant increase in the level/expression of the corresponding factor; ↓: significant decrease in the level/expression of the corresponding factor; →: indicates a causal or sequential relationship between two related factors in the proposed mechanism.

Despite offering new therapeutic perspectives for NPC invasion and metastasis, epigenetic therapies targeting non-coding RNAs still face substantial barriers to clinical translation, which can be broadly categorized into technical constraints and biological complexity.

At the technical level, the principal challenges for invasion-directed therapies are delivery efficiency and molecular stability. For instance, although antisense oligonucleotides (ASOs) targeting pro-invasive lncRNAs such as FAM225A can effectively silence their intended transcripts, heterogeneity within the tumor microenvironment may allow subsets of tumor cells to evade treatment (162). More importantly, miRNA mimics, including miR-873 mimics, are highly susceptible to nuclease-mediated degradation *in vivo* and exhibit limited penetration through dense tumor stroma, thereby restricting both their bioavailability and therapeutic duration (161). In the metastatic setting, therapeutic strategies are further constrained by the broad effects of epigenetic drugs. Although EZH2 inhibitors can restore miR-142-3p expression, their disruption of genome-wide H3K27me3 patterns may also impair normal cellular function (120).

At the biological level, the dynamic nature of non-coding RNA regulatory networks further complicates therapeutic intervention. For example, super-enhancer-derived RNAs (seRNAs) can drive metastasis through the hnRNPK/MICAL2 axis, yet their expression is dynamically governed by higher-order chromatin architecture, meaning that single-target intervention may provoke compensatory pathway activation (164). Likewise, replacement therapy involving the tumor-suppressive lncRNA EP300-AS1 requires precise control over its interaction with the transcription factor TFAP2C, a level of spatiotemporal regulation that current delivery platforms are not yet able to achieve reliably (163). Taken together, current approaches have not fully resolved the central tension between targeting precision and systemic controllability. Overcoming these limitations will require coordinated advances in both technological innovation and mechanistic understanding. First, intelligent delivery systems should be developed to improve therapeutic precision. Nanoparticles engineered to respond to tumor microenvironmental features, such as acidic pH or elevated matrix metalloproteinase activity, could be used to encapsulate ASO–miRNA complexes and enable controlled release specifically at lesion sites (160). In parallel, exosome-based delivery of EP300-AS1 may enhance lncRNA stability and tumor selectivity by exploiting the natural homing properties of exosomal carriers (163). Second, integration of epigenetic editing technologies may provide greater specificity than conventional epigenetic drugs. For example, the nonspecific effects of agents such as DNMT1 inhibitors could potentially be overcome by CRISPR/dCas9-based systems; fusion of dCas9 to a transcriptional activation domain and targeted delivery to the miR-142-3p promoter may enable locus-specific demethylation and transcriptional reactivation while avoiding the adverse consequences of global DNA modification (120). Similarly, base-editing approaches directed against seRNA-associated super-enhancer regions may allow precise suppression of their prometastatic activity (164). Finally, dynamic monitoring and feedback-guided treatment systems will be essential for addressing tumor evolution. Liquid biopsy-based tracking of CAF-derived miRNAs or circulating seRNAs could enable real-time assessment of therapeutic response and facilitate dose adjustment (165). Moreover, spatial mapping of lncRNA and miRNA expression across distinct tumor niches, such as the invasive front and hypoxic core, may identify region-specific targets and guide tailored delivery strategies. This concept is supported by recent single-cell and spatial transcriptomic studies that have uncovered mechanisms of radioresistance and immune evasion in recurrent NPC, highlighting the value of spatial expression profiling for the rational design of targeted RNA-based interventions (166). Combining artificial intelligence models to predict compensatory pathways after perturbation of non-coding RNA networks may help inform the design of multi-target sequential treatment regimens for more durable suppression of invasion and metastasis.

### 5.4 Current clinical translation: trials, approved epi-drugs, and biomarker implementation

To better anchor the translational discussion in concrete clinical examples, it is important to distinguish between three levels of evidence. First, the broader oncology field has already provided proof of principle that epigenetic therapy is clinically actionable, as illustrated by the regulatory approvals of azacitidine, decitabine/cedazuridine, and vorinostat in non-NPC malignancies (167). Second, clinical translation in NPC itself has begun to move beyond theoretical extrapolation. A phase II, multicenter, international, single-arm trial of oral azacitidine (CC-486; NCT02269943) enrolled previously treated patients with locally advanced or metastatic NPC using a Simon two-stage design. The study showed that oral epigenetic therapy was clinically deliverable and had manageable safety. However, the limited activity observed with single-agent treatment suggested that hypomethylating therapy alone is unlikely to provide sufficient efficacy in unselected recurrent or metastatic NPC. These findings suggest that, in NPC, epigenetic intervention may be more useful as a priming or sensitizing strategy within rational combinations than as a stand-alone treatment (6).

In the same translational direction, current NPC-specific trials are increasingly testing epigenetic agents in combination-oriented settings rather than as isolated monotherapies (168). For example, the decitabine-containing study NCT03701451 was designed to evaluate whether hypomethylating priming with decitabine plus cisplatin before concurrent chemoradiotherapy could enhance treatment sensitivity in regionally advanced NPC (169); notably, this trial used a Simon two-stage design and planned enrollment of approximately 30 patients, but mature efficacy data are not yet available (170). Likewise, the phase Ib/II study NCT07320963 is investigating chidamide in combination with toripalimab and anlotinib in recurrent/metastatic NPC, with an early focus on dose-limiting toxicity, maximum tolerated dose, and objective response rate. Although these studies do not yet provide practice-changing evidence, they illustrate an important shift in translational strategy: epigenetic therapy in NPC is increasingly being developed as a biomarker-informed combination platform intended to improve sensitivity to radiotherapy, immunotherapy, or antiangiogenic treatment (171). Third, biomarker implementation remains uneven: while plasma EBV DNA has already entered real-world clinical use, epigenetic biomarkers such as cfDNA methylation markers and EBV DNA methylation assays are still emerging (172). Therefore, the current clinical translation of NPC epigenetics is best viewed not as a completed transition to bedside application, but as an active shift from mechanistic proof-of-concept toward biomarker-guided, combination-based clinical development. Representative examples supporting the clinical translation of epigenetic strategies and biomarkers in NPC are compared in **Table 6**, which organizes the evidence according to category, clinical setting, translational relevance, and overall robustness. As shown in **Table 6**, the most mature clinically implemented evidence in NPC currently lies in biomarker application—especially plasma EBV DNA—whereas most epigenetic therapeutic approaches remain at the proof-of-concept, early translational, or trial-stage level.A practical roadmap for clinical implementation in NPC may be considered in three sequential steps. First, biomarker standardization should precede broad therapeutic deployment. This includes harmonized assay workflows, predefined positivity thresholds, and cross-cohort validation for methylation- and ncRNA-based markers, ideally benchmarked against the current clinical standard of plasma EBV DNA (187). Second, epigenetic agents should be prioritized in rational combination settings rather than empirically advanced as monotherapies. Based on current evidence, the most plausible near-term strategies are hypomethylating priming before chemoradiotherapy, epigenetic modulation combined with immune checkpoint blockade, and combination regimens that incorporate antiangiogenic therapy in recurrent/metastatic disease (188). Third, future trials should move toward biomarker-guided patient selection and mechanism-linked endpoints. Rather than relying only on conventional response assessment, studies should also evaluate whether epigenetic intervention achieves the intended biological effects, such as demethylation of target loci, restoration of immune-related transcriptional programs, or enhancement of treatment sensitivity in molecularly defined subgroups (188–190). In this sense, the most realistic translational path is not immediate replacement of current NPC treatment paradigms, but stepwise integration of validated biomarkers and mechanism-based epigenetic combinations into existing clinical workflows.

**Table 6:** Representative examples supporting the clinical translation of epigenetic strategies in nasopharyngeal carcinoma.

Category	Representative example	Clinical setting/current status	Translational relevance to NPC	Evidence robustness	References
Approved epigenetic drugs outside NPC	Azacitidine	FDA-approved in hematologic malignancies	Provides proof of principle that DNA hypomethylating therapy is clinically actionable, although direct efficacy in NPC remains to be established.	Moderate	(173, 174)
Approved epigenetic drugs outside NPC	Decitabine/cedazuridine	FDA-approved oral hypomethylating regimen in hematologic malignancies	Supports the feasibility of clinically deliverable DNMT-targeting strategies and offers a translational precedent for solid-tumor exploration.	Moderate	(175)
Approved epigenetic drugs outside NPC	Vorinostat	FDA-approved HDAC inhibitor	Demonstrates that histone-directed therapy can achieve regulatory approval and supports continued evaluation of HDAC-based approaches in NPC.	Moderate	(174, 176)
NPC-specific epigenetic trial	Oral azacitidine (CC-486; NCT02269943)	Phase II study in previously treated locally advanced or metastatic NPC	Supports prioritization of combination-based rather than monotherapy development in recurrent/metastatic NPC.	Moderate	(177, 178)
NPC-specific epigenetic trial	Decitabine-containing chemoradiotherapy (NCT03701451)	Registered NPC study	Represents a radiosensitization-oriented implementation path for locally advanced disease, pending outcome validation.	Preliminary	(179)
NPC-specific epigenetic trial	Chidamide plus toripalimab and anlotinib (NCT07320963)	Registered early-phase study in recurrent/metastatic NPC	Represents a combination-based implementation path for recurrent/metastatic disease, particularly in the context of immunotherapy-oriented development.	Preliminary	(180, 181)
Biomarker already used in practice	Plasma EBV DNA	Established clinical biomarker in NPC management	Remains the practical benchmark for screening, baseline risk stratification, treatment-response assessment, and surveillance.	Relatively strong	(182, 183)
Emerging epigenetic biomarker	Plasma cfDNA VILL methylation	Early clinical validation	Suggests that methylation-based liquid biopsy may complement EBV DNA testing for non-invasive detection.	Moderate	(184)
Emerging epigenetic biomarker	EBV DNA methylation in saliva or oropharyngeal swab	Early clinical validation	Indicates potential value for non-invasive triage and detection, but routine clinical implementation is not yet established.	Moderate	(185, 186)

## 6 Conclusion

Taken together, the evidence reviewed above indicates that epigenetic dysregulation is deeply involved in NPC invasion and metastasis and supports the concept of epigenetics as a “master switch” of malignant progression. Rather than simply cataloging abnormal epigenetic states, this review provides a metastasis-centered synthesis linking multilayer epigenetic regulation to EMT, tumor microenvironment remodeling, immune escape, therapy resistance, and clinically relevant biomarker and therapeutic implications. This review comprehensively examines epigenetic dysregulation in NPC invasion and metastasis and supports the concept of epigenetics as a “master switch” of malignant progression. Rather than simply cataloging epigenetic dysregulation, it provides a metastasis-centered synthesis linking multilayer epigenetic regulation to EMT, tumor microenvironment remodeling, immune escape, therapy resistance, and clinically relevant biomarker and therapeutic implications. To provide a broader view of NPC pathogenesis, we also integrate complementary evidence from proteomics, transcriptomics, and metabolomics. These studies highlight dysregulated phosphorylation networks, EMT-associated transcriptional programs, and metabolic reprogramming linked to epigenetic alterations such as SLC27A6 hypermethylation and ACAT1 promoter methylation. Overall, epigenetic regulation acts at an upstream level by reshaping chromatin accessibility and transcriptional states, thereby coordinating multiple downstream malignant processes rather than acting through a single isolated effector. This review also summarizes the prognostic significance of epigenetic markers and discusses emerging therapeutic strategies, including targeted inhibitors and epigenetic editing approaches. Although substantial progress has been made, clinical translation remains limited by biological heterogeneity and insufficient reproducibility of current detection and validation workflows. At present, plasma EBV DNA remains the benchmark biomarker in NPC management, whereas epigenetic biomarkers and epigenetic therapies are still advancing through early validation studies and NPC-specific trials toward biomarker-guided combination strategies.

## Glossary

ACAT1: Acetyl-CoA Acetyltransferase 1
AKT: Protein Kinase B
ALYREF: Aly/REF Export Factor
APC: Adenomatous Polyposis Coli
ARID1A: AT-Rich Interaction Domain 1A
ARNTL: Aryl Hydrocarbon Receptor Nuclear Translocator-Like
ASOs: Antisense Oligonucleotides
ATR: Ataxia Telangiectasia and Rad3-Related Protein
BAMBI: BMP and Activin Membrane-Bound Inhibitor
BRCA1: Breast Cancer 1
CAPN2: Calpain 2
CAFs: Cancer-Associated Fibroblasts
CBX1: Chromobox Homolog 1
CDK: Cyclin-Dependent Kinase
CDK1: Cyclin-Dependent Kinase 1
CDK4/6: Cyclin-Dependent Kinase 4/6
CDK5: Cyclin-Dependent Kinase 5
CLDN11: Claudin 11
CNE-1: C666–1 Nasopharyngeal Carcinoma Cell Line
CNE-2: CNE-2 Nasopharyngeal Carcinoma Cell Line
CST6: Cystatin E/M
CTTN: Cortactin
CyclinD1: Cyclin D1
dCas9: Dead CRISPR-Associated Protein 9
DDIT4: DNA Damage-Inducible Transcript 4
DGAT1: Diacylglycerol Acyltransferase 1
DNMT: DNA Methyltransferase
DNMT1: DNA Methyltransferase 1
DNMT2: DNA Methyltransferase 2
DNMT3A: DNA Methyltransferase 3A
DNMT3B: DNA Methyltransferase 3B
DNMT3L: DNA Methyltransferase 3-Like
DRAIC: Differentiation Antagonizing Non-Protein Coding RNA
EBV: Epstein-Barr Virus
EBF3: Early B-Cell Factor 3
ECRG4: Esophageal Cancer-Related Gene 4
EGFR: Epidermal Growth Factor Receptor
EMT: Epithelial-Mesenchymal Transition
EphA2: Ephrin Type-A Receptor 2
EP300-AS1: EP300 Antisense RNA 1
ERK: Extracellular Signal-Regulated Kinase
ERK1/2: Extracellular Signal-Regulated Kinase ½
E2F: E2F Transcription Factor
E1: Ubiquitin-Activating Enzyme
E2: Ubiquitin-Conjugating Enzyme
E3: Ubiquitin Ligase
FAK: Focal Adhesion Kinase
FAM225A: Family With Sequence Similarity 225 Member A
FBXL2: F-Box And Leucine Rich Repeat Protein 2
FTO: Fat Mass and Obesity-Associated Protein
FOXD1: Forkhead Box D1
FOXD1-AS1: FOXD1 Antisense RNA 1
FUS: Fused In Sarcoma
FZD10: Frizzled Class Receptor 10
GAS5: Growth Arrest-Specific 5
GATA1: GATA Binding Protein 1
GDF10: Growth Differentiation Factor 10
GSK-3β: Glycogen Synthase Kinase-3 Beta
GST: Glutathione S-Transferase
HATs: Histone Acetyltransferases
HDAC: Histone Deacetylase
HDAC2: Histone Deacetylase 2
HDAC4: Histone Deacetylase 4
HDAC7: Histone Deacetylase 7
HIF1α: Hypoxia-Inducible Factor 1 Alpha
HMGA2: High Mobility Group AT-Hook 2
HK2: Hexokinase 2
HOXA2: Homeobox A2
HOXA-AS2: HOXA Cluster Antisense RNA 2
HOPX: Homeodomain-Only Protein X
HR: Homologous Recombination
hnRNPK: Heterogeneous Nuclear Ribonucleoprotein K
HNE1: HNE1 Nasopharyngeal Carcinoma Cell Line
HONE1: HONE1 Nasopharyngeal Carcinoma Cell Line
ITGA6: Integrin Alpha 6
ITGB3: Integrin Beta 3
IOX1: Isoxazole-9
IPO7: Importin 7
JAK: Janus Kinase
JmjC: Jumonji C Domain-Containing Protein
KDM4A: Lysine Demethylase 4A
KLF5: Kruppel-Like Factor 5
KMTs: Lysine Methyltransferases
LATS1: Large Tumor Suppressor Kinase 1
LINC00173: Long Intergenic Non-Protein Coding RNA 173
LINC00839: Long Intergenic Non-Protein Coding RNA 839
LINC01503: Long Intergenic Non-Protein Coding RNA 1503
Lnc-MRPL39-2: 1, Long Non-Coding RNA MRPL39-2, 1
lncRNA: Long Non-Coding RNA
MAP7: Microtubule-Associated Protein 7
MAPK: Mitogen-Activated Protein Kinase
MBD: Methyl-CpG-Binding Domain
MBD1: Methyl-CpG-Binding Domain Protein 1
MBD2: Methyl-CpG-Binding Domain Protein 2
MBD3: Methyl-CpG-Binding Domain Protein 3
MBD4: Methyl-CpG-Binding Domain Protein 4
MECP2: Methyl-CpG-Binding Protein 2
METTL3: Methyltransferase Like 3
METTL14: Methyltransferase Like 14
MICAL2: MICAL Family Member 2
MMP-2: Matrix Metalloproteinase 2
MMP-9: Matrix Metalloproteinase 9
MKK3: Mitogen-Activated Protein Kinase Kinase 3
MLC2: Myosin Light Chain 2
MLCK: Myosin Light Chain Kinase
MFSD4A: Major Facilitator Superfamily Domain Containing 4A
miRNA: MicroRNA
miR-9-1: MicroRNA-9-1
miR-200: MicroRNA-200
miR-122: MicroRNA-122
miR-142-3p: MicroRNA-142-3p
miR-4465: MicroRNA-4465
miR-454-3p: MicroRNA-454-3p
miR-519: MicroRNA-519
miR-590-3p: MicroRNA-590-3p
miR-873: MicroRNA-873
miR-92b-3p: MicroRNA-92b-3p
miR-1275: MicroRNA-1275
m⁶A: N6-Methyladenosine
m⁵C: 5-Methylcytosine
mPFC: Medial Prefrontal Cortex
NF-κB: Nuclear Factor Kappa-Light-Chain-Enhancer of Activated B Cells
NFAT1: Nuclear Factor of Activated T Cells 1
Nectin-3: Nectin Cell Adhesion Molecule 3
NAD⁺: Nicotinamide Adenine Dinucleotide
NSUN: NOP2/Sun RNA Methyltransferase Family
NRXN3: Neurexin 3
NPC: Nasopharyngeal Carcinoma
Nuclear Smad2: Nuclear Mothers Against Decapentaplegic Homolog 2
p27: Cyclin-Dependent Kinase Inhibitor 1B
p38: p38 Mitogen-Activated Protein Kinase
p53: Tumor Protein P53
PA2G4: Proliferation-Associated Protein 2G4
PATs: Palmitoyltransferases
PCDH8: Protocadherin 8
PD-L1: Programmed Death-Ligand 1
PD1: Programmed Cell Death Protein 1
PHF2: PHD Finger Protein 2
PID: Protein-Protein Interaction Database
PI3K: Phosphoinositide 3-Kinase
PI3K/AKT: Phosphoinositide 3-Kinase/Protein Kinase B Signaling Pathway
PI3K-AKT-ERK: Phosphoinositide 3-Kinase-Protein Kinase B-Extracellular Signal-Regulated Kinase Signaling Pathway
PI3K/AKT/mTOR: Phosphoinositide 3-Kinase/Protein Kinase B/Mammalian Target of Rapamycin Signaling Pathway
PKC: Protein Kinase C
PKL: Polycomb Repressive Complex 1 Subunit
PWWP: PWWP Domain-Containing Protein
PXN: Paxillin
PRMTs: Protein Arginine Methyltransferases
PRMT1: Protein Arginine Methyltransferase 1
PRMT2: Protein Arginine Methyltransferase 2
PRMT3: Protein Arginine Methyltransferase 3
PRMT4: Protein Arginine Methyltransferase 4
PRMT6: Protein Arginine Methyltransferase 6
PRMT8: Protein Arginine Methyltransferase 8
PFS: Progression-Free Survival
RAD51: RAD51 Recombinase
ANKL: Receptor Activator of Nuclear Factor Kappa-B Ligand
RAS: Rat Sarcoma Viral Oncogene Homolog
RAB1A: RAB1A, Member RAS Oncogene Family
RAB1B: RAB1B, Member RAS Oncogene Family
Rb: Retinoblastoma Protein
RERG: RAS-Like Estrogen-Regulated Growth Inhibitor
Rhokinase: Rho-Associated Protein Kinase
ROCK: Rho-Associated Protein Kinase
ROCK1: Rho-Associated Protein Kinase 1
RKIP: Raf Kinase Inhibitor Protein
RTKs: Receptor Tyrosine Kinases
SAGE: Serial Analysis of Gene Expression
SAM: S-Adenosyl-L-Methionine
SATB1: Special AT-Rich Sequence-Binding Protein 1
SCD: Stearoyl-CoA Desaturase
SDF4: Stromal Cell-Derived Factor 4
SEPT9_v2: Septin 9 Variant 2
seRNAs: Super-Enhancer-Associated RNAs
SGSM1: Small G Protein Signaling Modulator 1
SIRT: Sirtuin
SLC27A6: Solute Carrier Family 27 Member 6
SNAIL: Snail Family Zinc Finger 1
Smad: Mothers Against Decapentaplegic Homolog
SNAIL: Snail Transcription Factor
Src: Src Proto-Oncogene, Non-Receptor Tyrosine Kinase
SRF: Serum Response Factor
SRA: Set and Ring Finger-Associated Domain
STAT1: Signal Transducer and Activator of Transcription 1
STAT3: Signal Transducer and Activator of Transcription 3
STING: Stimulator of Interferon Genes
TBP: TATA-Box Binding Protein
TDG: Thymine DNA Glycosylase
TET: Ten-Eleven Translocation Methylcytosine Dioxygenase
TFAP2C: Transcription Factor AP-2 Gamma
TGF-β: Transforming Growth Factor-Beta
TGF-β/Smad: Transforming Growth Factor-Beta/Mothers Against Decapentaplegic Homolog Signaling Pathway
TMEM130: Transmembrane Protein 130
TNFAIP2: TNF Alpha Induced Protein 2
TUBB3: Tubulin Beta 3 Class III
TUBA1B: Tubulin Alpha 1B
TEAD: TEA Domain Transcription Factor
THBS1: Thrombospondin 1
TRAF4: TNF Receptor-Associated Factor 4
TRIM21: Tripartite Motif Containing 21
TSC1: Tuberous Sclerosis Complex 1
UCHL1: Ubiquitin C-Terminal Hydrolase L1
UHRF: Ubiquitin-Like, Containing PHD and RING Finger Domains
UHRF1: Ubiquitin-Like, Containing PHD and RING Finger Domains 1
VDAC2: Voltage-Dependent Anion Channel 2
VEGF: Vascular Endothelial Growth Factor
Wnt/β-catenin: Wnt/Beta-Catenin Signaling Pathway
WTAP: Wilms Tumor 1-Associated Protein
ZEB2: Zinc Finger E-Box Binding Homeobox 2
ZIC2: Zinc Finger Protein of the Cerebellum 2
ZNF582: Zinc Finger Protein 582
αVβ3: Integrin Alpha V Beta 3
β-HB: Beta-Hydroxybutyrate
γH2AX: Phosphorylated Histone H2AX
5hmC: 5-Hydroxymethylcytosine
5mC: 5-Methylcytosine
5-8F: 5-8F Nasopharyngeal Carcinoma Cell Line
6-10B: 6-10B Nasopharyngeal Carcinoma Cell Line
S18: S18 Nasopharyngeal Carcinoma Cell Line
S26: S26 Nasopharyngeal Carcinoma Cell Line
SUNE1: SUNE1 Nasopharyngeal Carcinoma Cell Line
TGF-β/Smad: Transforming Growth Factor-Beta/Mothers Against Decapentaplegic Homolog Signaling Pathway
AKT/Wee1/CDK: Protein Kinase B/Wee1/Cyclin-Dependent Kinase Signaling Pathway
EGFR/AKT/ERK: Epidermal Growth Factor Receptor/Protein Kinase B/Extracellular Signal-Regulated Kinase Signaling Pathway
JAK/STAT: Janus Kinase/Signal Transducer and Activator of Transcription Signaling Pathway
GST: Glutathione S-Transferase
MTDH: Metadherin
PID: Protein-Protein Interaction Database
SAGE: Serial Analysis of Gene Expression
SCD: Stearoyl-CoA Desaturase
TSC1: Tuberous Sclerosis Complex 1
γH2AX: Phosphorylated Histone H2AX
VEGF: Vascular Endothelial Growth Factor
